# Pre-Colonization of B*acillus siamensis* on Ocular Surface Mitigates F*usarium keratitis* Through Direct Antifungal Activity and Pre-Activation of NF-κB Pathway

**DOI:** 10.1167/iovs.66.12.38

**Published:** 2025-09-17

**Authors:** Xudong Zhao, Zhichao Ren, Dingwen Cao, Zheng Shao, Min Liu, Yusen Huang

**Affiliations:** 1 State Key Laboratory Cultivation Base, Shandong Key Laboratory of Eye Diseases, Shandong Eye Institute, School of Ophthalmology, Shandong First Medical University, Qingdao, People's Republic of China; 2 The Second School of Clinical Medicine, Xuzhou Medical University, Xuzhou, People's Republic of China; 3 Qingdao Yusen Eye Hospital, Qingdao, China

**Keywords:** ocular surface microbiome, *Bacillus siamensis* (*B. siamensis*), fungal keratitis (FK), NF-κB pathway

## Abstract

**Purpose:**

Fungal keratitis (FK) is a severe ocular disease that leads to corneal ulceration and permanent vision loss. The ocular surface microbiota comprises beneficial symbiotic and pathogenic bacteria. Therefore, this study aimed to isolate beneficial bacterial strains from the ocular surface and evaluate their effect on *Fusarium* infections.

**Methods:**

Alterations in the ocular surface microbiota of patients with FK were analyzed using 16S rRNA sequencing. Candidate bacteria were isolated from mouse eyeballs and evaluated for antifungal activity. Quantitative PCR (qPCR) and culture were used to determine the colonization efficiency of *Bacillus siamensis* (*B. siamensis*). In addition, its biological safety was assessed. The effects of *B. siamensis* pre-colonization on *Fusarium keratitis* were evaluated using slit-lamp examination, clinical scoring, optical coherence tomography, hematoxylin and eosin staining, and RNA sequencing. Western blotting and RT-qPCR were used to assess its effects of on NF-κB signaling and inflammation, whereas flow cytometry was used to measure changes in immune cell populations following *B. siamensis* pre-colonization.

**Results:**

Ocular surface microbiota of patients with FK had significantly low levels of *Bacilli*. *B. siamensis* exhibited significant direct antifungal activity with minimal toxicity. Pre-colonization with *B. siamensis* mitigated FK-associated corneal edema and opacity, structural damage, inflammation, and fungal burden. Additionally, it enhanced ocular surface immunity into a “pre-immune” state by activating NF-κB pathway in healthy mice cornea.

**Conclusions:**

*B. siamensis* on the ocular surface can directly engulf *Fusarium* hyphae and secrete antifungal substances to exert antifungal effects. Moreover, it enhanced ocular surface immunity into a “pre-immune” state by activating NF-κB pathway to facilitate rapid immune response.

Fungal keratitis (FK) is a severe ocular infection characterized by corneal ulceration and inflammation. If left untreated, FK can lead to serious complications, including corneal perforation and intraocular infection. Globally, the annual incidence of FK is estimated to range from 736,251 to 1,367,323 cases.^1^ Despite treatment, the prognosis of FK remains concerning, with approximately 10% affected eyes requiring enucleation or experiencing perforation, and over 60% patients developing monocular blindness.^1^^,^^2^ These statistics underscore the critical need to improve the clinical prognosis of this potentially sight-threatening disease.^3^^,^^4^

The clinical prognosis of FK is largely determined by the interplay between virulence and host defense mechanisms.^5^ Host defense can be categorized into two main components: physical barrier protection and immune response.^5^^,^^6^ The ocular surface is protected by a diverse array of anti-inflammatory and antimicrobial substances conferring innate immunity, primarily found in the tear fluid, which inhibit or eliminate microbial threats. These include secretory IgA, mucin glycoproteins, and antimicrobial peptides.^5^^,^^7^ Concurrently, the innate immune system recognizes conserved components of fungal cell walls, such as chitin, β-glucan, and mannan, which act as pathogen-associated molecular patterns (PAMPs).^5^ The PAMPs are detected by pattern recognition receptors (PRRs) expressed on various immune cells, including macrophages, dendritic cells, and neutrophils. The interactions between PAMPs and PRRs facilitate pathogen adhesion, absorption, and subsequent eradication, thereby playing a crucial role in host defense mechanisms against fungal infections.^5^^,^^8^ Thus, the optimization of the local immune microenvironment may improve the clinical prognosis of patients with FK.

The ocular surface microenvironment shares similarities with the skin, possessing innate microbiota and prolonged direct exposure to external environmental factors.^9^ A delicate equilibrium is maintained within this ecosystem via intricate interactions among the local microbiota, immune substances, immune cells, and ocular cells. Any disruption in this balance may lead to infection. The local microbiota also includes probiotics, which may maintain local immune homeostasis and resist external pathogens^10^^–^^12^—with examples including the genera *Bacillus*, *Lactobacillus*, and *Bifidobacterium*.^13^^,^^14^ To date, *Corynebacterium mastitidis* is the only bacterium known to have an ocular effect, primarily inducing IL-1β production by activating ocular γδ T cells.^15^ However, research on the role of ocular surface microbiota in maintaining local immune homeostasis, resisting external pathogens, and its relationship with specific eye diseases remains limited.

Pathogenic fungi can swiftly infiltrate corneal tissues and secrete toxic factors that compromise corneal barrier function. Concurrently, resident immune cells in the cornea produce inflammatory mediators and facilitate neutrophil recruitment to combat fungal invasion. However, the excessive release of these mediators and over-recruitment of neutrophils often exacerbate corneal tissue damage.^16^^,^^17^ Therefore, an optimal approach to managing FK involves suppressing fungal growth while balancing host immune activation to minimize inflammation-related tissue injury.^18^ In this regard, the complex interactions among the host immune system, fungal pathogens, and the microbiota, along with multiple regulatory mechanisms encompassing direct and indirect antifungal effects, may contribute to reducing tissue damage and preventing excessive inflammation.^19^^–^^22^

In this study, we successfully isolated bacterial strains that could counter fungal infections and identified them to be *Bacillus siamensis (B. siamensis)*. Subsequent experiments indicated that *B. siamensis* can successfully colonize the mouse ocular surface and enhances ocular surface immunity into a “pre-immune” state by activating the NF-κB pathway. Upon infection with *Fusarium*, mice colonized with this strain showed stronger resistance and significantly reduced inflammatory responses compared with non-colonized mice.

## Materials and Methods

### Clinical Sample Collection and 16S rRNA Sequencing

Conjunctival swab samples from affected and healthy eyes of patients with FK (detailed information was described in the Supplementary Methods) were collected using sterile swabs soaked in oxybuprocaine hydrochloride eye drops in an operating room conforming to the GB50333-2013-I standard. The sterile swabs, along with the remaining oxybuprocaine hydrochloride eye drops, were sent to OE Biotech (Qingdao, China) for DNA extraction using a DNA Extraction Kit (Omega Bio-tek, Norcross, GA, USA) followed by processing for high-throughput 16S rRNA sequencing. The detailed 16S sequencing methodology is described in the Supplementary Methods.

### Animals

Adult C57BL/6 mice with a wild-type (WT) genetic background were acquired from Beijing Vital River Laboratory Animal Technology (Beijing, China). The experimental animals were maintained in a specific pathogen-free environment. All experimental procedures followed the international standards outlined in the ARVO Statement for the Use of Animals in Ophthalmic and Vision Research. The study protocol was approved by the Animal Investigation Committee of the Eye Institute of Shandong First Medical University (approval number: SDSYKYJS No. 20230219).

### Cultivation of *Fusarium solani*

The *Fusarium solani* strain (AS 3.1829) was sourced from the China General Microbiological Culture Collection Center (CGMCC, Beijing, China) and cultured on PDA medium at 28°C for 5 to 7 days. The spore concentration of the *Fusarium solani* (*F. solani*) suspension was then adjusted to 1 × 10⁸ spores/mL using PBS for subsequent use.

### Candidate Strain Screening and its Enzyme Preparation

C57BL/6 mice were euthanized, and their eyeballs and conjunctival swabs were collected, homogenized in 2 mL of sterile PBS, and plated onto Gause’s No. 1 agar (Hope Bio-technology, Qingdao, China). After incubation at 37°C for 48 hours, individual colonies were selected and cultured in lysogeny broth (LB) medium. Bacterial suspensions were co-cultured with *F.* *solani* spore suspensions (1 × 10⁸ spores/mL in PBS) at a 10:1 volume ratio at 37°C for 36 hours. After centrifugation, the supernatant was filtered through a 0.22 µm membrane to obtain the crude enzyme solution. The preparation protocol for the ocular surface transient crude enzyme solution is described in the Supplementary Methods.

The antifungal activity of the crude enzyme solution was evaluated by applying 50 µL of the solution to PDA plates pre-inoculated with *F. solani*. Plates were incubated at 37°C for 72 hours, and hyphal degradation was observed. The strain with the strongest antifungal activity was selected and identified by 16S rRNA sequencing (Tsingke Biotechnology, Beijing, China).

### Antifungal Activity of *B. siamensis* Against *F. solani*

To further evaluate antifungal potential, PDA plates were uniformly spread with *F. solani* spores, and a sterile filter paper disc soaked in 1 × 10⁸ CFU/mL *Bacillus siamensis* suspension was placed at the center. After incubation at 27°C for 48 hours, the extent of the inhibition zone was measured using ImageJ software (NIH, Bethesda, MD, USA).

Additional experimental procedures, including spore germination inhibition assays and Gram staining-based microscopic analysis of *B. siamensis–F. solani* dual-culture confrontation, are described in the Supplementary Methods.

### Cell Viability Assay

The viability of corneal epithelial cells (detailed information is described in the Supplementary Methods) was assessed using the CCK-8 assay (Beyotime, Shanghai, China). Cells were co-cultured with serial dilutions (1:5–1:200) of *B. siamensis* crude enzyme solution for 24 hours and tested as per the manufacturer’s instructions.

### *B. siamensis* Inoculation and Quantification

Mice were anesthetized with isoflurane,^15^^,^^23^ and the conjunctiva was dabbed with a sterile cotton swab to disrupt the tear film. The preparation and application of materials for bacterial colonization on the ocular surface using the GelMA system are described in the Supplementary Methods.^24^^–^^27^ The GelMA-mediated colonization approach was performed daily for 7 consecutive days. After the 7-day colonization process was completed, tear fluid, conjunctival swabs, and corneas were collected and homogenized in sterile PBS. Thereafter, 100 µL of each dilution was plated on Bacillus-selective agar and incubated at 37°C for 24 hours to count CFUs. DNA was extracted using the FastPure Host Removal and Microbiome DNA Isolation Kit (Vazyme, Nanjing, China) and used for quantitative real-time polymerase chain reaction (qRT-PCR).

### Corneal Safety Assay

Alterations in the cornea were examined during the colonization phase using slit-lamp microscopy and fluorescein sodium staining to assess corneal safety. Upon completion of colonization, a 2.5 mm epithelial defect was created to evaluate corneal wound healing. Fluorescein sodium staining was performed at 24 and 36 hours, followed by observation under cobalt blue light to monitor healing. The residual defect area was subsequently quantified using ImageJ software.

### *Fusarium keratitis* Mouse Models

FK was induced in C57BL/6 mice using a validated protocol adapted from previous studies.^18^^,^^28^^,^^29^ Three days after completion of *B. siamensis* colonization, the mice were anesthetized via intraperitoneal injection of 0.6% sodium pentobarbital (0.25–0.3 mL), and topical ocular anesthesia using oxybuprocaine hydrochloride eye drops. A central corneal epithelial defect, approximately 2.5 mm in diameter, was created using an electric scraper under a surgical microscope. A sterile soft contact lens (5 mm) was placed on the injured cornea, and 5 µL of the *F. solani* spore suspension (1 × 10⁸ CFU/mL) was applied between the wound and the lens. The eyelids were sutured with 7-0 silk to maintain the contact lens position and facilitate infection. The suture and lens were carefully removed after 24 hours.

### Clinical Scoring

FK progression was monitored daily via slit-lamp biomicroscopy and graded according to a validated 12-point clinical scale.^30^ This system quantifies disease severity using three criteria: corneal opacity area, stromal infiltration density, and epithelial surface integrity, each assigned a score of 0 (normal) to 4 (severe) pathology.

### Corneal Thickness Measurements

Central corneal thickness was analyzed using anterior segment OCT (RTVe XR 100; Opteva, Inc., Fremont, CA, USA) equipped with a dedicated corneal adaptor lens (maximum scan depth = 2.4 mm and scan width = 8.0 mm). Sagittal cross-sectional images were acquired in crossline mode, and the ruler tool provided with the instrument was used to calculate the corneal thickness.

### Hematoxylin and Eosin Staining

After fixation, the eyeballs were embedded in paraffin. Serial corneal sections (prepared using an ultramicrotome) were subjected to hematoxylin and eosin (H&E) staining and examined under a microscope (Eclipse E800) for histopathological evaluation.

### Corneal Fungal Load Assay

The fungal burden was quantified 3 days post-infection. Corneas were homogenized in PBS, serially diluted, and plated on potato dextrose agar (PDA) plates. After incubation at 37°C, CFUs were enumerated, with the final load calculated by multiplying colony counts by the corresponding dilution factor.

### Immunofluorescence Staining

Ocular specimens were cryoembedded in optimal cutting temperature compound (Sakura, Torrance, CA, USA) and sectioned at 7-µm thickness. Following fixation with 4% paraformaldehyde (SigmaAldrich, St. Louis, MO, USA) and permeabilization with 0.5% Triton X-100, sections were incubated with anti-p65 primary antibody (ab32536, 1:100 dilution; Abcam, Cambridge, UK) at 4°C overnight. After washing with PBS, the sections were stained with an Alexa Fluor 488–conjugated Goat anti-Rabbit IgG secondary antibody (Thermo Fisher Scientific, Waltham, MA, USA) for 2 hours. Nuclei were counterstained with DAPI and images were acquired using a Revolve fluorescence microscope (Echo, San Diego, CA, USA).

### Quantitative Real-Time Polymerase Chain Reaction

Total RNA was isolated using a commercial RNA extraction kit (TransGen Biotech, Beijing, China), followed by cDNA synthesis using reverse transcriptase (Vazyme). The qRT-PCR amplification was performed on an ABI Prism 7500 platform (Applied Biosystems, Waltham, MA, USA) using ChamQ Universal SYBR qPCR Master Mix (Vazyme). β-ACTIN was utilized as an internal reference, and the relative expression levels were analyzed using the 2^−ΔΔCt^ method. The primer sequences used are listed in the Table.

**Table.** Primer Sequences in RT-qPCR

Genes	Forward Primer (5′–3′)	Reverse Primer (5′–3′)
TNF-α	TGATGACATCAAGAAGGTGGTGAAG	TCCTTGGAGGCCATGTGGGCCAT
IL-1β	GAAATGCCACCTTTTGACAGTG	TGGATGCTCTCATCAGGACAG
IL-6	TAGTCCTTCCTACCCCAATTTCC	TTGGTCCTTAGCCACTCCTTC
Bssc-16S	TGGCTTCGGCTACCACTTACA	GCTGCCTCCCGTAGGAGTCT
β-actin	GGCTGTATTCCCCTCCATCG	CCAGTTGGTAACAATGCCATGT

### Western Blotting

Corneal tissues were homogenized in ice-cold RIPA lysis buffer (Solarbio, Beijing, China) containing 1% phenylmethylsulfonyl fluoride (PMSF) and a phosphatase inhibitor cocktail (CWBio, Jiangsu, China). Protein lysates were resolved by 10% sodium dodecyl sulfate-polyacrylamide gel electrophoresis) and electrotransferred to PVDF membranes (EMD Millipore, Billerica, MA, USA). After blocking with 5% BSA (Solarbio), the membranes were probed with primary antibodies overnight at 4°C, followed by incubation with horseradish peroxidase-conjugated goat anti-rabbit secondary antibodies. Protein bands were detected using ECL substrate (EMD Millipore) and imaged using the ChemiDoc Touch System (Bio-Rad, Hercules, CA, USA). Relative quantification was performed by densitometry image analysis using ImageJ software. Antibody specifications are listed in Supplementary Table S1.

### Flow Cytometry

Mouse corneal tissues, including epithelium, stroma, and endothelium, were carefully dissected for single-cell preparation. The whole corneas were pretreated with dispase II (15 mg/mL, 4°C, for 24 hours) to facilitate tissue separation. Subsequently, the epithelial and endothelial layers were enzymatically dissociated using trypsin, while the stromal layer was digested separately with collagenase (Roche, Basel, Switzerland). Resulting cell suspensions were stained with fluorochrome-conjugated antibodies under light-protected conditions at 4°C for 30 minutes, including FITC anti-CD45 (30-F11), APC anti-CD3 (17A2), and PerCP/Cyanine5.5 anti-CD19 (6D5; all from BioLegend, San Diego, CA, USA). FSC-A and SSC-A gating strategies were used to exclude debris, and doublets were identified by plotting FSC-A versus FSC-H. Cells were analyzed using a FACSCalibur flow cytometer (CytoFLEX; Beckman Coulter, Fullerton, CA, USA), and data were processed using CytExpert and FlowJo 10.0 software.

### RNA Sequencing and Analysis

Corneal tissues were collected from two groups of mice: one group that was pre-colonized with *B. siamensis* prior to *Fusarium keratitis* infection, and another group infected with *Fusarium keratitis* alone. After enucleation of the eyeballs, the corneas were carefully dissected and excised under a stereomicroscope. The isolated corneas were immediately snap-frozen in liquid nitrogen and stored at −80°C until further processing. The frozen samples were sent to Personal Biotechnology (Shanghai, China) for total RNA extraction and transcriptome (RNA-seq) analysis. The transcriptomic analysis methods are described in detail in the Supplementary Methods.

### Statistical Analysis

All data analyses were performed using the GraphPad Prism 9 software (GraphPad Software, San Diego, CA, USA). All experiments were conducted with at least three biological replicates, and the data are presented as mean ± standard deviation (SD). Statistical comparisons between the two groups were performed using an unpaired two-tailed Student's *t*-test, whereas one-way analysis of variance with the Tukey post hoc test was used for multiple group comparisons. Statistical significance was set at *P* < 0.05.

## Results

### 16S rRNA Sequencing Revealed the Significantly Under-Represented Abundance of Bacillus on the Ocular Surface of the Affected Eye in Patients With FK 

**Figure 1.**
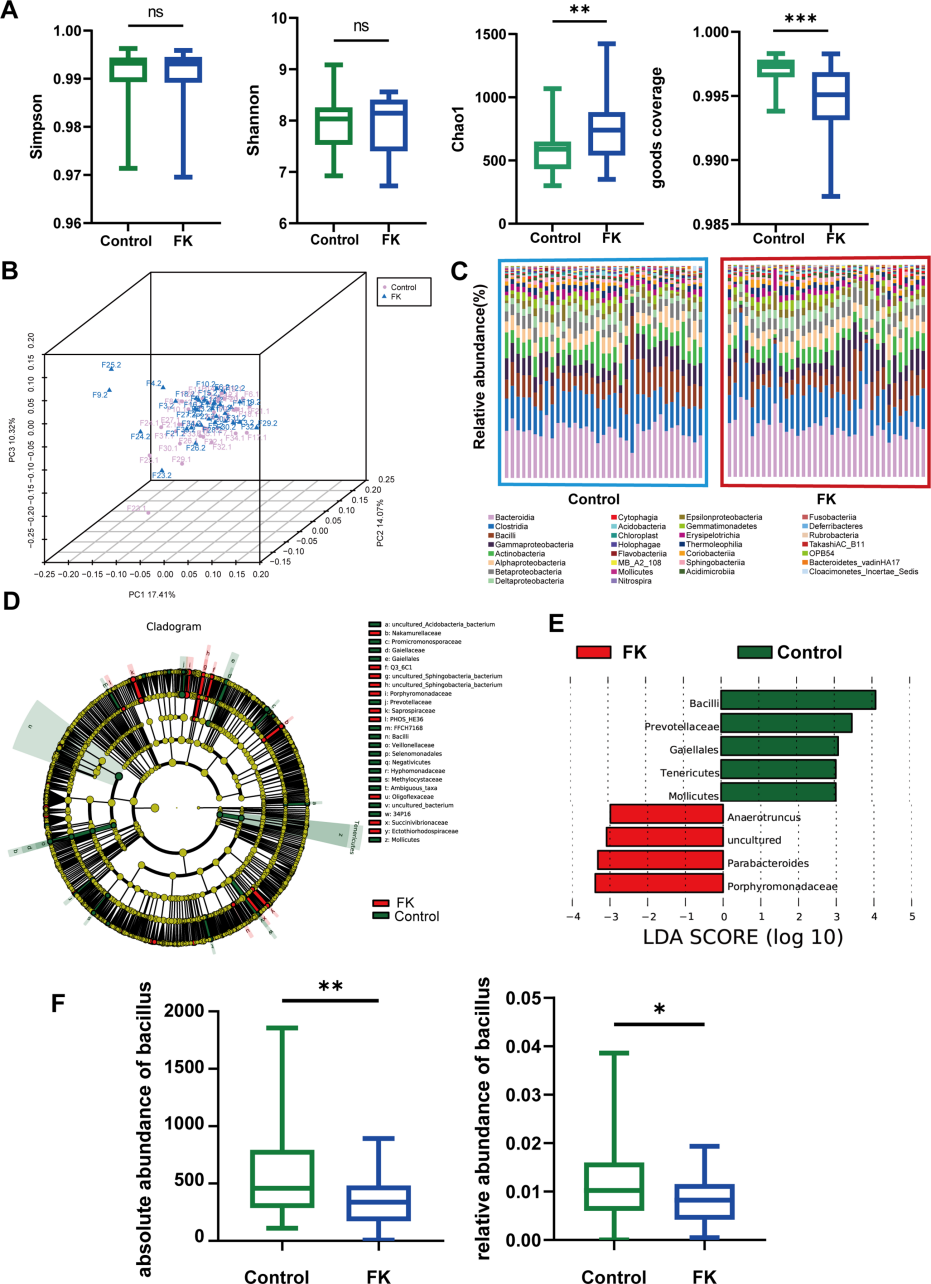
**Ocular surface microbiota characteristics between healthy and FK subjects.** (**A**) Alpha diversity indexes (*n* = 35). (**B**) Beta diversity (**n** = 35). (**C**) The composition of ocular surface microbiota in each sample visualized using top 30 enriched genus (**n** = 35). (**D,** **E**) LEfSe analysis. (**F**) Absolute and relative abundances of *Bacillus* genus (*n* = 34 and *n* = 32). Not significant = *P* > 0.05; * *P* < 0.05; ** *P* < 0.01; *** *P* < 0.001.

We performed 16S rRNA sequencing of the ocular surface microbiota from the affected eyes and healthy eyes of patients with FK to evaluate FK-related alterations in the ocular surface microbiota, specifically the alpha and beta diversity indices. Although the Simpson and Shannon indices did not present significant differences between the two groups, the Chao1 and Goods coverage indices indicated significant differences (Fig. 1A). Additionally, the two groups showed significant differences in beta diversity (Fig. 1B). The composition of the ocular surface microbiota in each sample was visualized using the top 30 most enriched genera (Fig. 1C). An LEfSe analysis was performed to identify the significantly differential taxa in the ocular surface microbiota from patients with FK (Figs. 1D, 1E). At multiple taxonomic levels, *Bacillus* was under-represented in FK-affected eyes, whereas *Anaerotruncus* and *Parabacteroides* were upregulated. We previously hypothesized that an imbalance between protective and pathogenic bacteria in the ocular microbiota of healthy individuals may trigger susceptibility to infection.^31^ Thus, this downregulated genus in FK-affected eyes may contain protective bacteria. Further comparison (Fig. 1F) suggested that the relative and absolute abundances of *Bacillus* genus were significantly under-represented in FK-affected eyes compared with healthy controls. These findings suggest a potential role for *Bacillus* in the pathophysiology of FK.

### *B. siamensis* Isolated From the Ocular Surface Exhibits In Vitro Antifungal Activity Against *F. solani*

**Figure 2.**
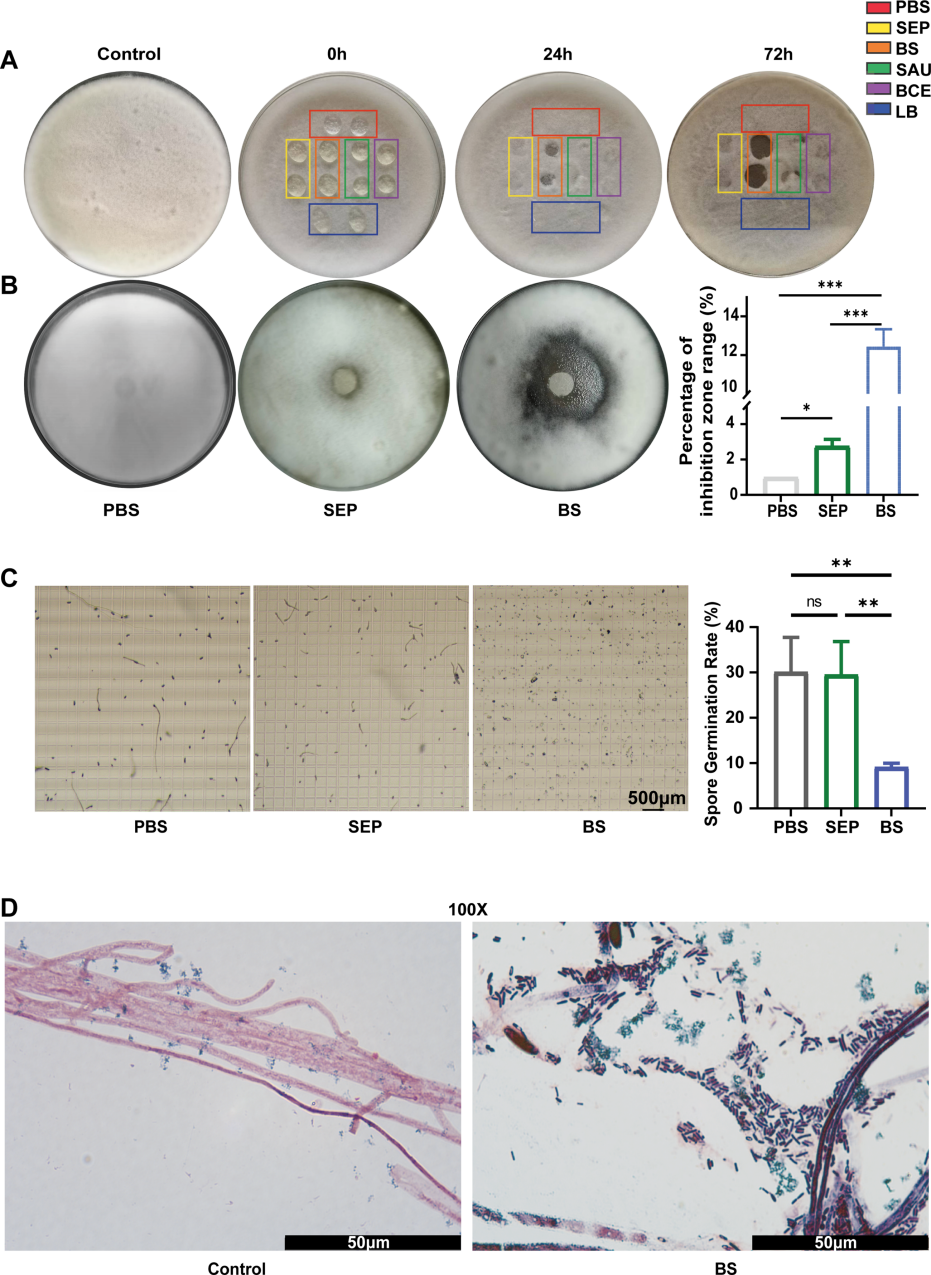
***B. siamensis* isolated from the ocular surface exhibits in vitro antifungal activity against *F. solani*.** (**A**) Antifungal activity, which is visualized by hyphal dissolution zone, bacterial strains isolated from the ocular surface of WT C57BL/6 mice. SEP, *Staphylococcus epidermidis*; SAU, *Staphylococcus aureus*; BCE, *Bacillus cereus*; BS, *B. siamensis*; PBS, phosphate buffered saline; LB, lysogeny broth (*n* = 6). (**B**) Inhibition zone of agar diffusion assay of *Staphylococcus epidermidis* and *B. siamensis* (*n* = 3). (**C**) Inhibition rate of spore germination of *Staphylococcus epidermidis* and *B. siamensis* (*n* = 3). (**D**) Microscopic analysis of the co-culture of *B. siamensis* and *F. solani* (*n* = 3). Not significant = *P* > 0.05; * *P* < 0.05; ** *P* < 0.01; *** *P* < 0.001.

We isolated bacterial strains from the ocular surface of WT C57BL/6 mice and verified their antifungal activity by observing their capacity for fungal hyphal dissolution against *F. solani* to screen for potential antifungal genera. The results identified 12 bacterial strains, including *Staphylococcus epidermidis*, *Staphylococcus aureus*, *Bacillus cereus*, *Escherichia coli*, *Streptococcus viridans*, and *B. siamensis* (Supplementary Table S2)*.* Among them, *B. siamensis* displayed the most potent antifungal activity, producing the largest hyphal dissolution zones (Fig. 2A). Additionally, *B. siamensis* exhibited remarkable inhibitory effects on spore germination and fungal growth (Figs. 2B, 2C). Furthermore, Gram staining analysis of the *B. siamensis* and *F. solani* co-culture showed that *B. siamensis* significantly attenuated fungal invasion, as indicated by substantially fragmented fungal structures in the *B. siamensis* pre-colonization group compared with the healthy controls (Fig. 2D). These results highlight the antifungal activity of *B. siamensis*.

### Application of *B. siamensis* Does not Alter Corneal Clarity or Wound Healing Dynamics

**Figure 3.**
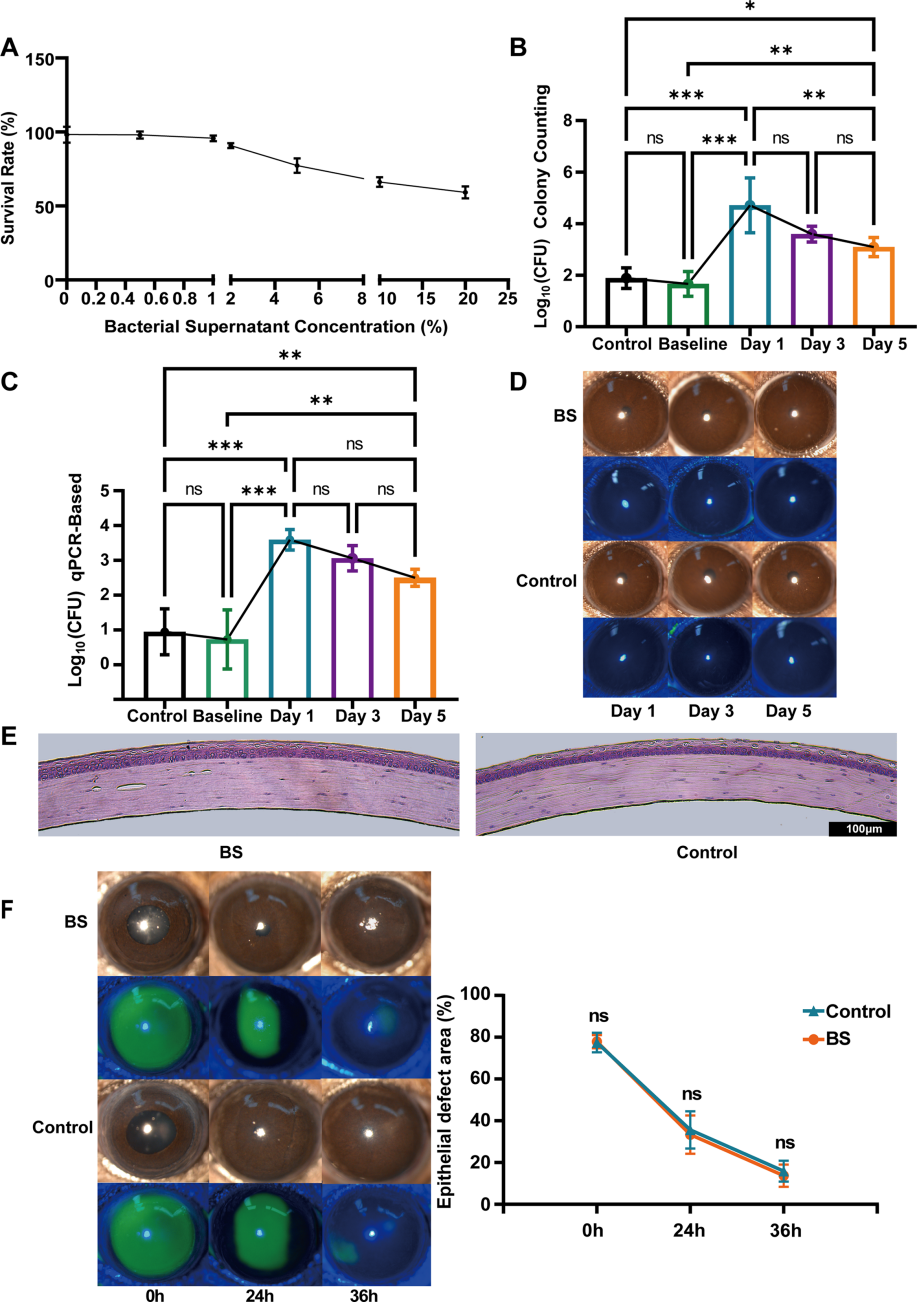
**Application of *B. siamensis* does not alter corneal clarity or wound healing dynamics.** (**A**) Dose-dependent epithelial cell survival analysis with increasing concentrations of *B. siamensis* crude enzyme solutions (*n* = 3). (**B**) Bacterial load in the *B. siamensis* (BS) colonized group and control group (received blank GEL photocrosslinking) quantified by RT-qPCR at the baseline group (baseline before colonization), day 1, day 3, and day 5 (*n* = 5). (**C**) Bacterial load quantified by culture-based assay following *B. siamensis* colonization (*n* = 3). (**D**) Slit-lamp images showing the ocular surface with both normal slit-lamp examination and fluorescein sodium staining in the BS group and the control group (*n* = 6). (**E**) H&E-stained histological images in the BS group and control group following colonization completion on day 3 (*n* = 3). *Scale bar* = 100 µm. (**F**) Sodium fluorescein staining and epithelial defect healing measured (*n* = 6). Not significant = *P* > 0.05; * *P* < 0.05; ** *P* < 0.01; *** *P* < 0.001.

Before evaluating the in vivo potential of *B. siamensis* against *Fusarium*-induced FK, we used a CCK-8 assay to test the cytotoxicity of its metabolites in corneal epithelial cells. The results indicated that these metabolites did not significantly reduce cell viability at low to moderate concentrations (≤ 2.0%), maintaining survival rates above 90% (Fig. 3A). However, a decline in cell viability was observed starting at a concentration of 5% of the crude enzyme solution from *B. siamensis*, suggesting that *B. siamensis* metabolites begin to exert cytotoxic effects at this threshold. Subsequently, we used 1 × 10^8^ CFU of *B. siamensis* and assessed colonization performance. The detection of *B. siamensis* colonization began after the completion of the colonization process, as indicated by cultural and qPCR results; its abundance peaked on the first day after colonization (day 1) and then gradually declined. However, the bacterial count stabilized at a relatively consistently low level thereafter (Figs. 3B, 3C). *B. siamensis* did not induce corneal opacification, edema, or signs of infection in the colonization model (Fig. 3D). Histological examination using H&E staining revealed no inflammatory cell infiltration (Fig. 3E). Moreover, *B. siamensis* did not delay healing of the corneal epithelium (Fig. 3F). Similar to the colonization of *B. siamensis*, we also attempted to colonize *S. aureus* and *S. epidermidis* on the ocular surface. The results showed that *S. aureus* colonization triggered inflammatory responses, whereas *S. epidermidis* appeared relatively safe (Supplementary Figs. S1A, S1B). These findings demonstrate that *B. siamensis* is safe during colonization and that ocular microbiota safety is strain dependent.

### *B. siamensis* Colonization Alleviates the Severity of FK

**Figure 4.**
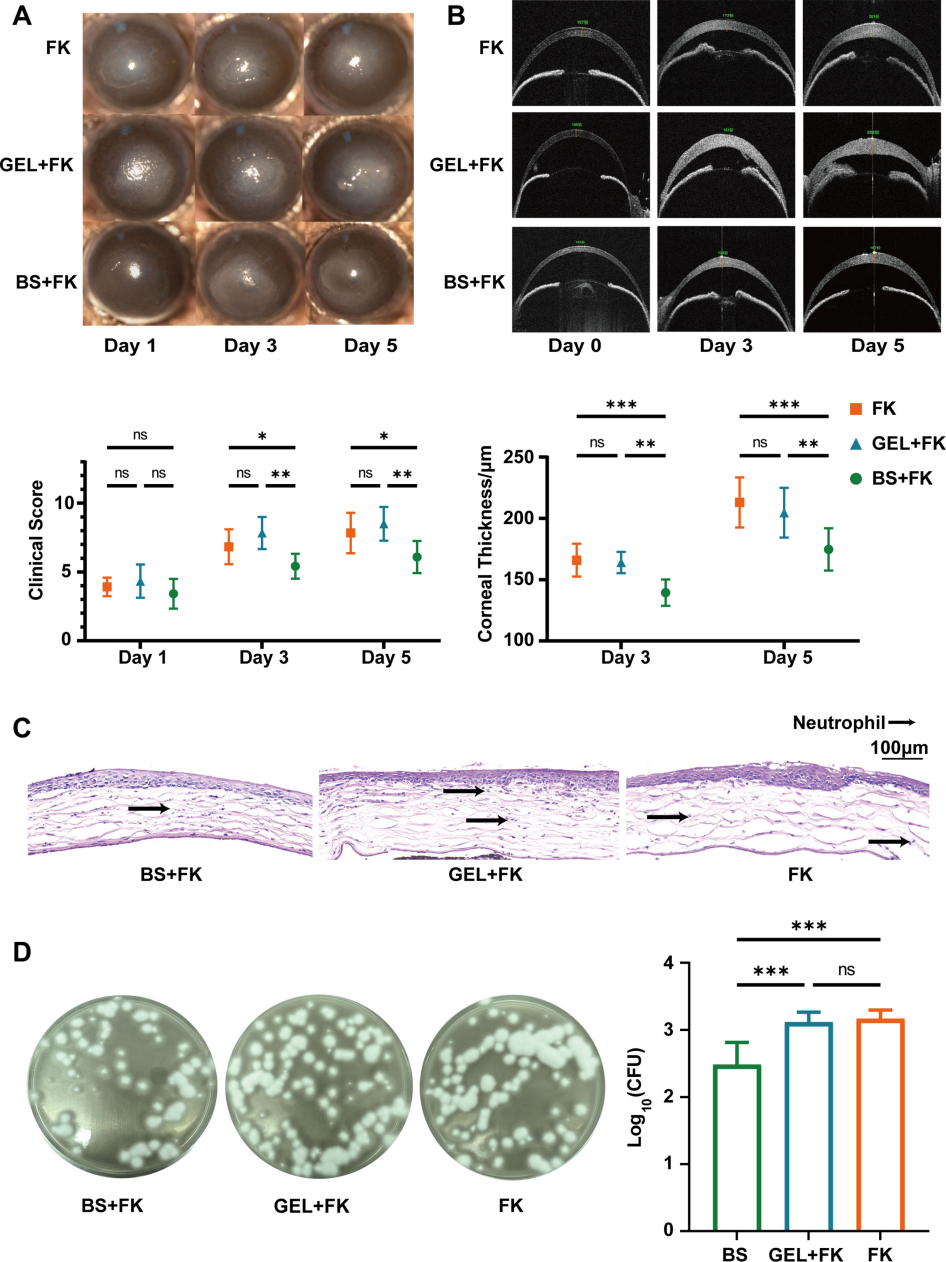
***B. siamensis*** **colonization** **alleviates the** **severity of FK.** (**A**) Slit-lamp images showing corneal opacification and inflammation in the pre-colonized with *B. siamensis* (BS + FK, *n* = 12), blank material control group receiving photocrosslinked material (GEL + FK, *n* = 6) and untreated fungal keratitis control group (FK; *n* = 12). Clinical inflammation scores compare the severity of inflammation. (**B**) The anterior segment optical coherence tomography (AS-OCT) images and corneal thickness measurements (FK, *n* = 12; GEL + FK, *n* = 6; BS + FK, *n* = 12). (**C**) H&E-stained histological images showing inflammatory cell infiltration and stromal structure (*n* = 3). *Scale bar* = 100 µm. (**D**) Fungal burden analysis with representative plate culture images (*left*) and fungal CFU quantification (*right*) (*n* = 6). Not significant = *P* > 0.05; * *P* < 0.05; ** *P* < 0.01; *** *P* < 0.001.

Corneal opacification, clinical scores, and corneal edema in the FK mouse model, including the FK group, GEL + FK group (treated with blank hydrogel), progressively worsened from day 1 to day 5. Conversely, mice pre-colonized with *B. siamensis* (BS + FK) showed significantly alleviated corneal opacification, clinical scores, and corneal edema on days 3 and 5 (Figs. 4A, 4B). Histopathological examination further revealed that the *B. siamensis* colonized group maintained a notably more intact corneal architecture (Fig. 4C). Additionally, CFU assays revealed that the fungal burden in the corneas of *B. siamensis*-colonized mice exhibited a reduction compared to the other groups (Fig. 4F). These results suggested *B. siamensis* colonization can alleviates the severity of FK, which may relate to its inhibiting effect on fungal burden.

### *B. siamensis* is Rapidly Depleted in the Ocular Surface Microbiota During the Fight Against *Fusarium* Infection

Based on the sequencing results of the human ocular surface microbiota, we noted that *B. siamensis* is depleted after fungal infection, which may render it unable to continuously exert antifungal effects during the pathological progression of FK. To maintain the stability of *B. siamensis* abundance, we planned to intervene via topical ocular application. Given the potential risks of directly dropping live bacteria during FK,^32^^,^^33^ we chose to administer a crude enzyme solution (S. CE) derived from the post-colonization ocular surface microbiota of mice, attempting to restore the ocular surface microenvironment through this solution. Quantitative analysis of ocular surface *B. siamensis* at 3 days post-infection revealed a significant decrease in *B. siamensis* abundance in both the FK group and the BS + FK group, and instillation of S. CE failed to maintain its abundance (Supplementary Fig. S2A). The tests showed that the enzyme solution exerted no antifungal activity in vitro (Supplementary Fig. S2B). Meanwhile, the S. CE group did not alleviate the clinical symptoms of FK (Supplementary Figs. S2C, S2D). After infection is established, the decline in *B. siamensis* abundance renders it unable to participate continuously in antifungal defense, suggesting that it primarily exerts a preventive role rather than being involved throughout the entire pathological process of FK.

### RNA-Seq Analysis Suggests *B. siamensis* Colonization Downregulated Inflammatory Signaling Pathways in FK Corneal Tissue

**Figure 5.**
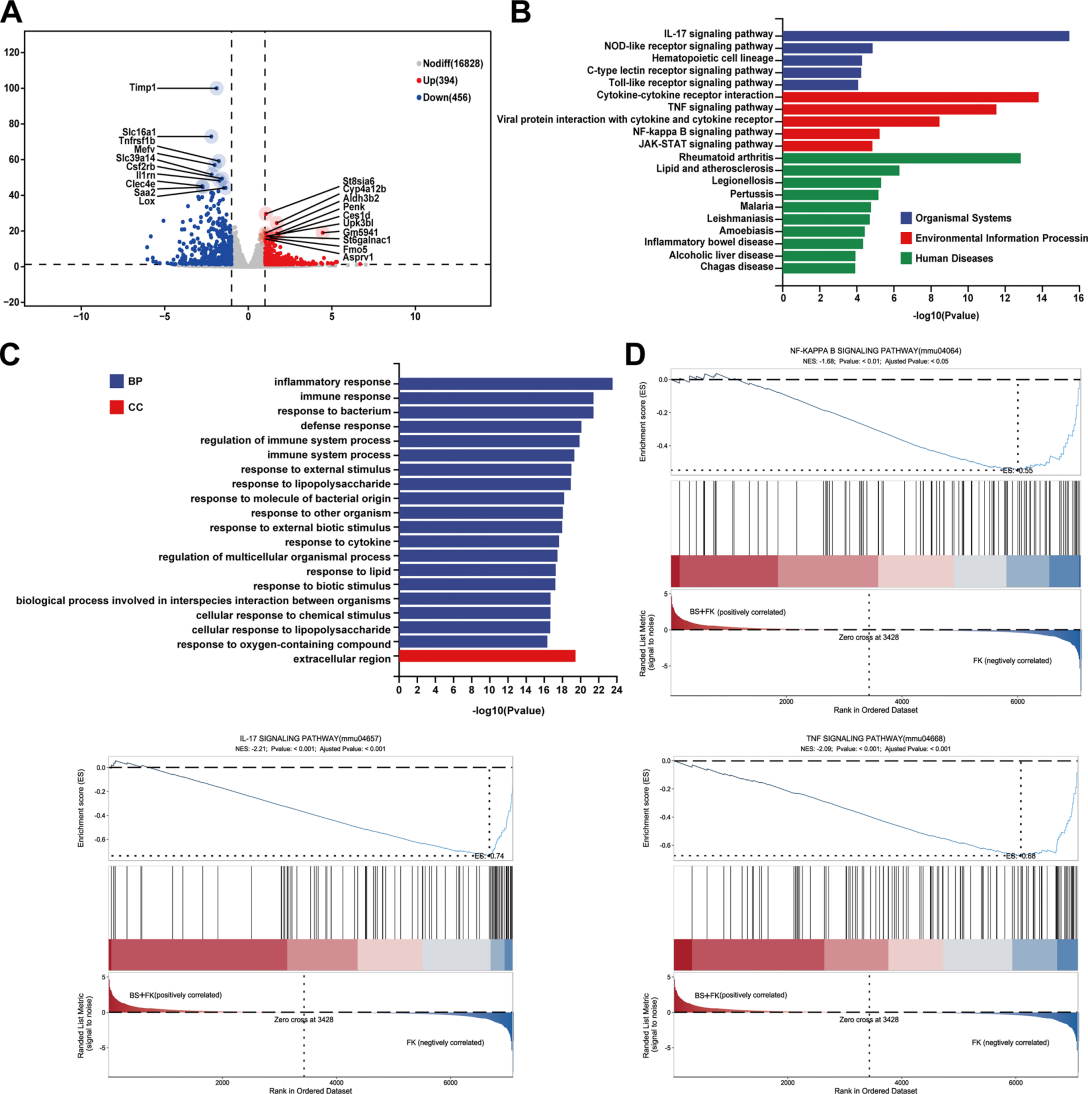
**RNA-seq analysis suggest *B. siamensis*** **colonization downregulated** **inflammatory signaling pathways in FK corneal tissue.** (**A**) Volcano plot of differentially expressed genes (DEGs) comparing the *B. siamensis* treatment (BS + FK) and the FK groups, showing upregulated genes (*red*), downregulated genes (*blue*), and non-differentially expressed genes (*gray*). (**B**) KEGG pathway enrichment analysis identifying significantly enriched immune-related pathways. (**C**) GO enrichment analysis showing biological processes related to inflammatory response, immune response, and response to bacterial stimuli in DEGs. (**D**) GSEA plots of IL-17, TNF, and NF-κB signaling pathways. The ES < 0 and ES peaks appear in the BS + FK negative correlation region (*blue area*), indicating significant downregulation of the enriched gene set.

We conducted RNA-seq analysis to compare the corneal transcriptomic profiles of *B. siamensis* colonized and uncolonized mice following *Fusarium* infection and elucidate the molecular mechanisms underlying the protective effects of *B. siamensis* colonization against FK. PCA revealed notable differences in gene expression profiles between FK corneas (the FK group) and corneas pre-colonized with *B. siamensis* (the BS + FK group; Supplementary Fig. S3A). Our initial examination revealed 243 upregulated and 287 downregulated genes in the pre-colonized group (Fig. 5A). The protein-protein interaction (PPI) network analysis of the differentially expressed gene interactions includes a series of immune-related proteins (Supplementary Fig. S3B). Gene Ontology (GO) enrichment analysis of differentially expressed genes revealed several immune- and inflammation-related biological processes (Figs. 5B, 5C). The Kyoto Encyclopedia of Genes and Genomes (KEGG) pathway analysis revealed the activation of key immune signaling cascades, most notably the IL-17, TNF, and NF-κB pathways. This pronounced reduction in pro-inflammatory gene expression suggests that *B. siamensis* may help temper hyper-inflammatory responses during *Fusarium* infection.

Next, we performed Gene Set Enrichment Analysis to comprehensively assess the differentially expressed pathways (Fig. 5D). The results suggested that NF-κB (NES = –1.68), IL-17 (NES = –2.21), and TNF (NES = –2.09) are crucially involved in orchestrating the antifungal activities of the cornea. RNA-seq and pathway analysis indicate that pre-colonization with *B. siamensis* significantly reduces the expression of pro-inflammatory genes, such as IL-17, TNF, and NF-κB in *Fusarium keratitis*, potentially through inhibition of fungal growth.

**Figure 6.**
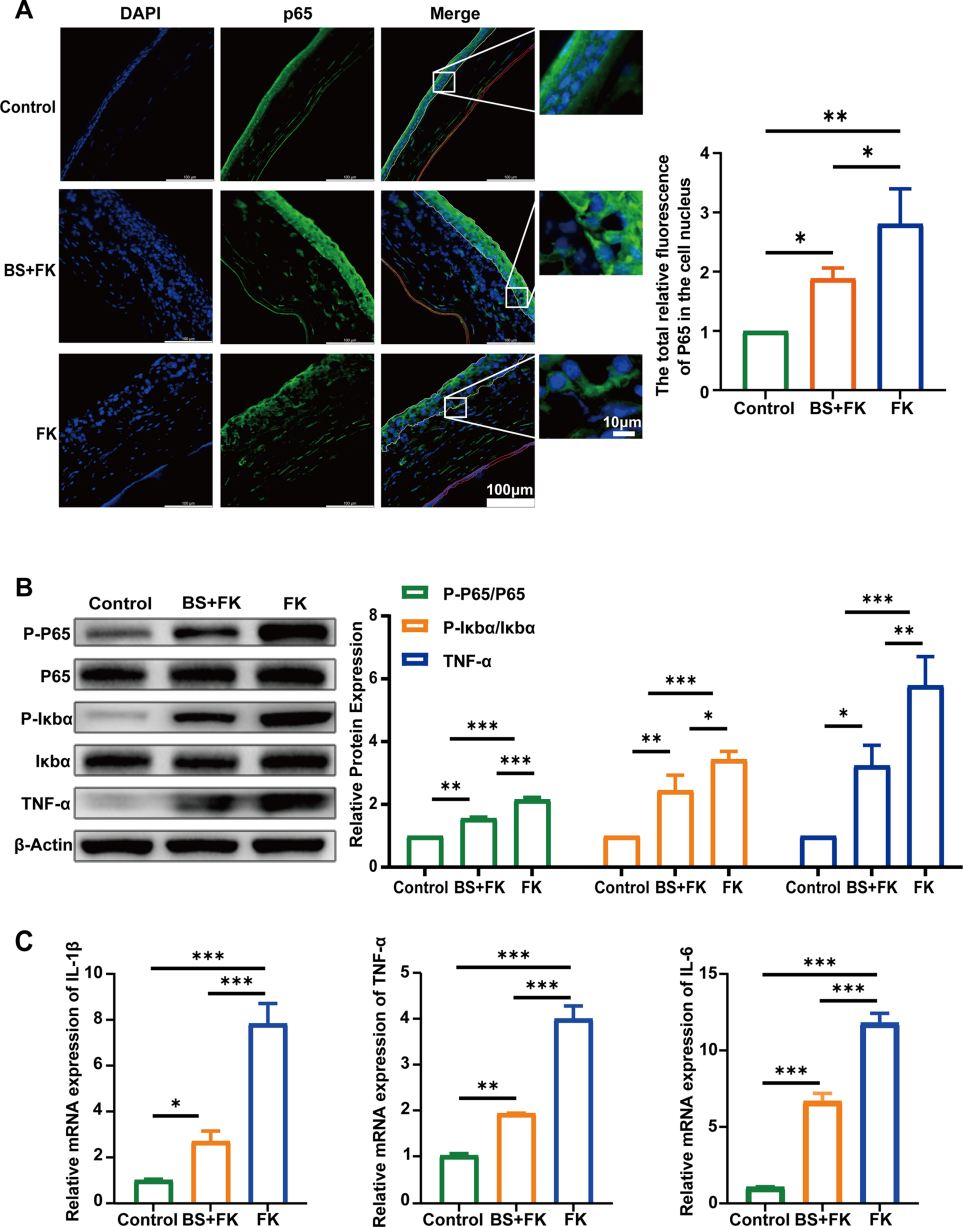
**Direct** **fungicidal** **action of *B.* *siamensis*** **curtails NF-κB–mediated** **inflammation in *Fusarium keratitis*.** (**A**) Immunofluorescence staining of p65 showing its localization (*green*) in the BS + FK, FK, and control groups (received blank GEL photocrosslinking), with DAPI (*blue*) marking the nucleus. The corneal epithelium and endothelium were outlined by *white* and *red lines*, respectively, and global quantification of nuclear p65 fluorescence intensity was performed (*n* = 3). Representative regions with nuclear translocation of p65 were magnified. (**B**) Western blot analysis of protein expression of P-p65, P-IκBα, TNF-α, and IκBα in the BS + FK, FK, and control groups, with β-actin as a loading control. Protein quantification of P-p65/p65, P-IκBα/IκBα, and TNF-α levels in all groups (*n* = 3). (**C**) The mRNA expression analysis showing relative expression of IL-1β, TNF-α, and IL-6 (*n* = 3). * *P* < 0.05; ** *P* < 0.01; *** *P* < 0.001.

The direct antifungal activity of *B. siamensis* may contribute to the downregulation of inflammatory pathways. Given that the NF-κB pathway acts as a key downstream effector of both IL-17 and TNF signaling, we focused on investigating the impact of *B. siamensis* on the NF-κB pathway. Immunofluorescence assays showed nuclear translocation of the key NF-κB subunit p65 in all groups except the control group, indicating activation of the NF-κB signaling pathway and its involvement in the inflammatory process (Fig. 6A). Subsequent protein-level analyses demonstrated that the ratios of p-p65/p65 and p-IκBα/IκBα were significantly lower in the pre-colonized group compared with the FK group. Additionally, TNF-α protein expression followed the same trend (Fig. 6B). Additionally, neither the blank material nor crude enzyme solution exerted a significant effect on NF-κB signaling pathway in the infection group (Supplementary Fig. S4). The mRNA levels of the inflammatory cytokines IL-1β, IL-6, and TNF-α were significantly upregulated in FK mice but were markedly lower in the pre-colonized group (Fig. 6C). In view of the inhibiting effect of *B. siamensis* pre-colonization on fungal burden in mice FK model, *B. siamensis* pre-colonization diminishing the infection-driven inflammatory response and consequently downregulating the NF-κB signaling pathway mainly due to the directly antifungal effect.

### *B. siamensis* Colonization Enhances Ocular Surface Immunity into a “Pre-Immune” State

**Figure 7.**
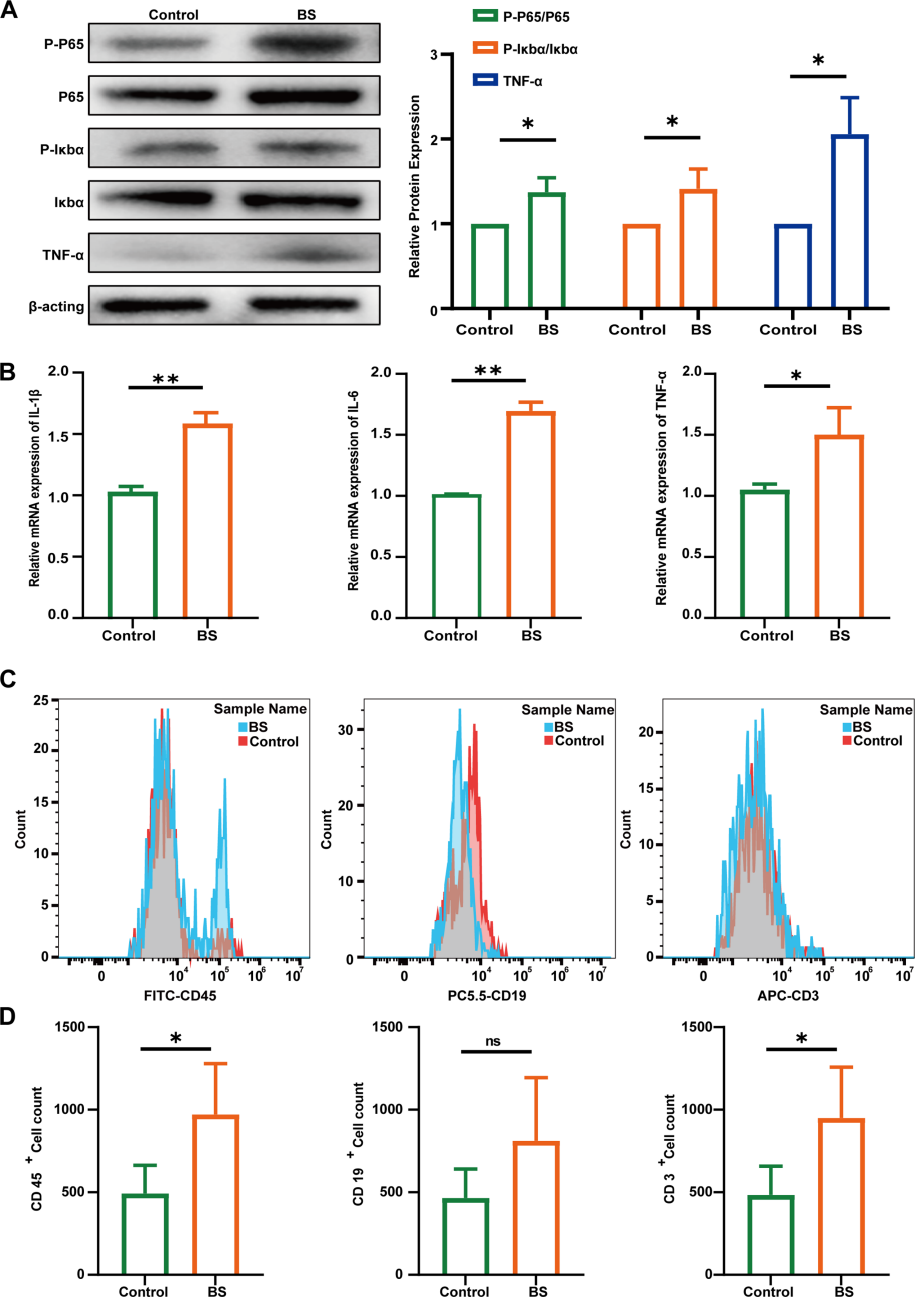
***B. siamensis* colonization enhances ocular surface immunity into a “pre-immune” state by activating** **the** **NF-κB pathway.** (**A**) Western blot analysis of protein expression of P-p65, P-IκBα, p65, TNF-α, and IκBα in the *B. siamensis* and control groups (received blank GEL photocrosslinking), with β-actin as a loading control. Protein quantification of P-p65/p65, P-IκBα/IκBα, and TNF-α levels in all groups (*n* = 3). (**B**) Relative mRNA expression levels of inflammatory cytokines IL-1β, IL-6, and TNF-α (*n* = 3). (**C**) Flow cytometry analysis of CD45^+^ cells, CD3^+^ T cells, and CD19^+^ B cells in the BS and control groups. (**D**) Quantification of CD45^+^, CD3^+^, and CD19^+^ cell counts in the BS and control groups (*n* = 3). Not significant = *P* > 0.05; * *P* < 0.05; ** *P* < 0.01.

**Figure 8.**
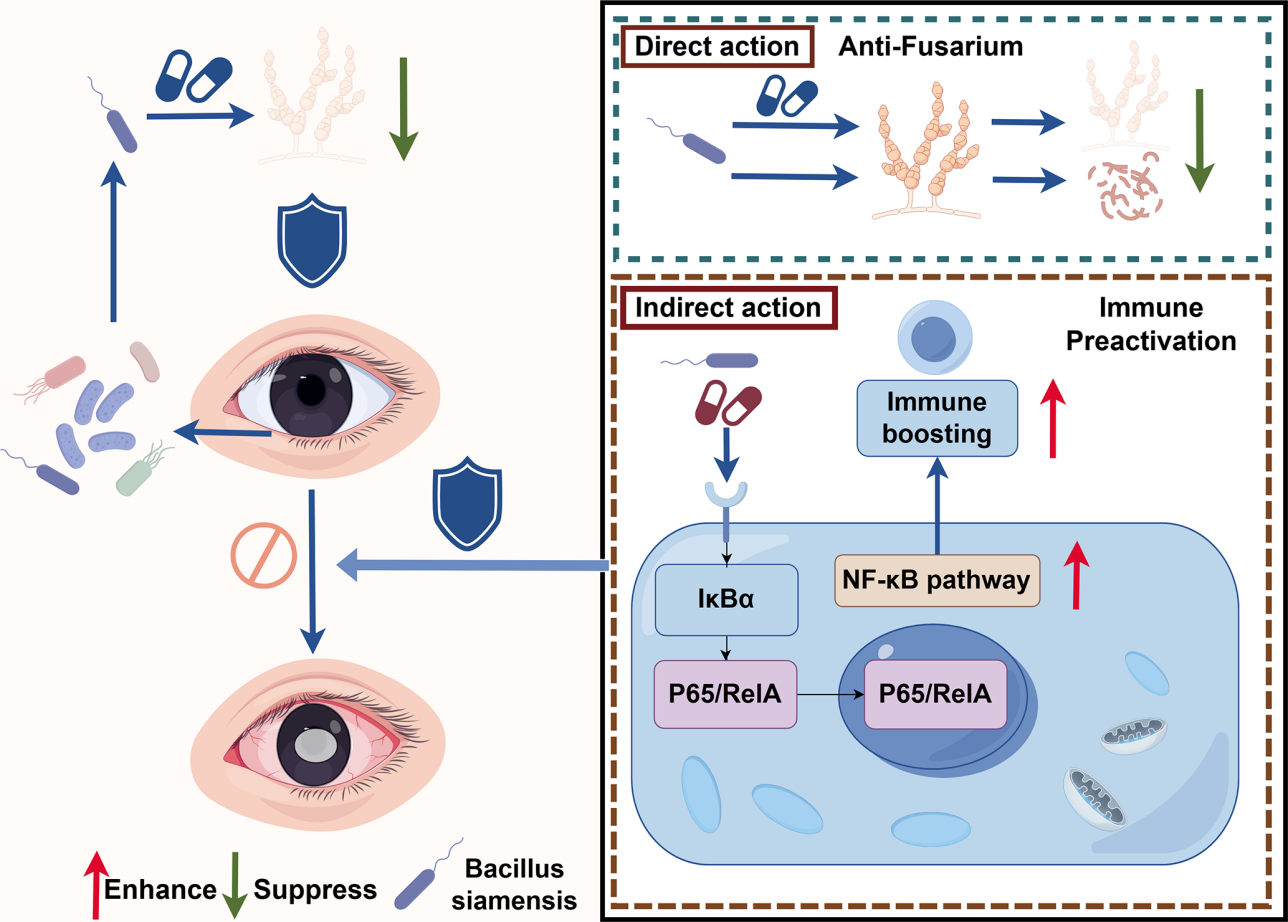
**Schematic representation of the effects of *B. siamensis* on *Fusarium* in FK.** *B. siamensis* directly engulfs *Fusarium* hyphae, secretes antifungal substances, and occupies specific ecological niches on the ocular surface. Additionally, it exhibited regulation of the host NF-κB signaling pathway by pre-activating, thereby enhancing ocular surface immunity to a “pre-immune” state.

We compared NF-κB pathway activation between the *B. siamensis* colonized group and the control group to ascertain the effect of *B. siamensis* on normal ocular surface. The NF-κB signaling pathway was activated by *B. siamensis* colonization (Fig. 7A), resulting in upregulation of mRNA levels of IL-1β, IL-6, and TNF-α with *B. siamensis* colonization (Fig. 7B). Additionally, flow cytometry analysis showed a mild increase in CD3⁺ T cell infiltration in the cornea of mice colonized with *B. siamensis*, whereas CD19⁺ B cell levels remained unchanged (Fig. 7C, 7D). These findings suggest that *B. siamensis*-mediated regulation of NF-κB activation may confer a “pre-immune” state, enabling a more rapid response to true pathogenic invasion while minimizing pathological damage. Subsequently, NF-κB activation was assessed in the SEP group to ascertain whether this “pre-immune” phenomenon is unique to *B. siamensis* and independent of conserved bacterial components. *B. siamensis* alone mildly activated this pathway, whereas SEP colonization did not (Supplementary Fig. S5). *B. siamensis* gently primes NF-κB, establishing a protective “pre-immune” tone that is not evident in SEP controls. During FK, the presence of *B. siamensis* is associated with a moderated NF-κB phosphorylation profile, a pattern that coincides with reduced fungal burden (Supplementary Fig. S6). Together with *B. siamensis*’ capacity for direct antifungal activity in vitro and its pre-immunization mechanism, these observations suggest a multifaceted contribution summarized in Figure 8 (drawn using Figdraw).

## Discussion

In this study, we isolated and identified a strain of *B. siamensis* from the ocular surface of mice that exhibited notable antifungal activity against *Fusarium*. We systematically evaluated its capacity to inhibit fungal growth, reduce pathogen burden, and alleviate tissue damage in vitro and in vivo. Notably, this strain demonstrated multifaceted protective mechanisms: it directly engulfs *Fusarium* hyphae, secretes antifungal substances, occupies specific ecological niches,^34^^–^^36^ and pre-activate the host NF-κB signaling pathway. Consequently, *B. siamensis* accelerates the immune response to pathogen invasion, thereby reducing the fungal burden and mitigating excessive inflammatory damage to the cornea.

Previous studies of the ocular surface microbiome have primarily focused on compositional differences across various disease conditions using high-throughput sequencing technology. Zheng Shao et al.^37^ identified ocular microbiota differences between patients with cataracts with and without type 2 diabetes. Fu et al. described the metagenomic profile of ocular surface microbiome changes in patients with Demodex blepharitis.^38^ Irina Schlegel et al.^39^ reported on the human ocular surface microbiome characteristics in dry eye disease. Livia Spörri et al.^40^ examined the ocular surface microbiome in patients with glaucoma. However, these studies primarily presented sequencing results of the ocular surface microbiome composition without elucidating the underlying mechanisms responsible for these differences, such as the cause and effect of alterations.

In light of this, we previously^41^ analyzed the ocular surface microbiome composition and found that patients with bacterial keratitis had significantly fewer beneficial symbiotic bacteria but a higher prevalence of common ocular pathogenic bacteria compared with healthy individuals. Consequently, we hypothesized that an imbalance between protective and pathogenic bacteria in the ocular microbiota of healthy individuals may increase their susceptibility to keratitis. The present study builds on these previous 16S sequencing findings by conducting further research. Our findings identified *Bacillus* as the bacterial genus that was significantly reduced on the ocular surface during FK infection. Our results suggest that *B. siamensis* can inhibit pathogen overgrowth and modulate local immune responses, indicating that it plays a distinct functional role in the ocular surface microbiota.

*B. siamensis*, a Gram-positive bacterium belonging to the phylum Firmicutes, exhibits unique biocontrol properties. This bacterium produces cyclic lipopeptides and secretes extracellular hydrolytic enzymes, thereby inhibiting the growth and biofilm formation of various pathogenic fungi, including *Fusarium* species.^42^^,^^43^ Our in vitro observations of the fungistatic effects of *B. siamensis* against *Fusarium* spp. are consistent with these mechanisms and explain the mechanism by which it occupies ecological niches upon colonization and reduces fungal loads.^34^^–^^36^ Pedretti et al.^44^ isolated a *B. siamensis* strain from human skin and demonstrated that its cell-free supernatant (CFS) inhibited multiple pathogenic microorganisms, while exhibiting resistance to protease degradation and high temperatures. Similarly, our study found that the crude enzyme (CE) of *B. siamensis* exhibited direct antifungal activity in vitro, confirming its intrinsic antifungal potential. In contrast, the crude enzyme preparation derived from the ocular surface microbial community after *B. siamensis* colonization (S. CE) did not exhibit antifungal activity after in vitro amplification. This was attributed to the failure of in vitro culture to maintain the ocular surface microecological structure, which also led to its inability to sustain the ocular surface abundance of *B. siamensis*. These findings suggest further research on in vitro cultivation of ocular surface microbial communities is needed. Additionally, Rungsirivanich et al.^45^ found that this organism displayed high viability and adhesion in simulated gastric and intestinal fluids, which may partially explain its stable colonization of the ocular surface. To the best of our knowledge, this is the first study to report the beneficial effects of *B. siamensis* on the ocular surface, thus expanding our understanding of its potential applications in ocular health and disease management.

The NF-κB signaling pathway plays a crucial role in driving host anti-infective immune responses in FK by regulating pro-inflammatory cytokines.^16^^,^^46^ Recent interventions for FK have increasingly focused on modulating NF-κB signaling. Several compounds have shown promise in regulating the NF-κB pathway through various mechanisms, effectively alleviating inflammation and promoting tissue repair. For instance, Ebselen and Dimethyl Fumarate (DMF) activate the Nrf2 pathway and upregulate antioxidant proteins such as HO-1, thereby indirectly inhibiting NF-κB and reducing the production of pro-inflammatory cytokines like IL-1β and TNF-α.^18^^,^^47^ In contrast, tetramethylpyrazine (TMP) directly inhibits TLR4/MyD88/NF-κB signaling and NLRP3 inflammasome activation, consequently mitigating inflammation.^48^ Accordingly, this approach represents a promising strategy for the management of FK by addressing the infectious and inflammatory aspects of the disease.

In this study, colonization of *B. siamensis* in a *Fusarium*-infected mouse model significantly reduced inflammation. This is consistent with Fang and Liu who indicate that *B. siamensis* can modulate NF-κB or MAPK signaling in mouse colonic cells and in the intestinal cells of the spotted sea bass (Lateolabrax maculatus), significantly reducing pro-inflammatory factors such as TNF-α and IL-6 while upregulating anti-inflammatory molecules, including IgA and immunoglobulin A.^49^^,^^50^ The microbiome–immune signaling regulation observed in this study is consistent with the above results. We also discovered that under healthy conditions, *B. siamensis* can moderately pre-activate NF-κB, providing a baseline for immune defense. This defense is promptly amplified when pathogens invade, which is consistent with the mechanisms identified in our *Fusarium* infection model. This regulatory effect of *B. siamensis* on the NF-κB pathway represents a novel mechanism by which commensal bacteria may contribute to ocular health. A previous study on *Corynebacterium mastitidis* in a murine model demonstrated that this bacterium can induce the production of IL-1β by activating ocular γδ T cells, thereby promoting sIgA secretion and exerting immunomodulatory effects.^15^^,^^51^ Conversely, no sIgA was detected in the eyes of germ-free mice. These observations imply the existence of a unique ecological selection pressure on the ocular surface that may be harnessed for future probiotic development in the ocular environment.

Despite these promising findings, this study has some limitations that warrant consideration. First, the full spectrum of beneficial symbiotic bacteria on the ocular surface has not been comprehensively reported owing to potential limitations in bacterial adaptation to the culture medium used.^52^ Second, as this study primarily involved healthy mice, the efficacy of *B. siamensis* in various pathological conditions, such as immunodeficiency, dry eye syndrome, and meibomian gland dysfunction, remains to be verified. Third, the cause of the decline in *B. siamensis* abundance following the onset of FK remains unclear, which may partially account for its limited prophylactic efficacy. Finally, the long-term ecological impact of *B. siamensis* colonization on the ocular surface microbiome and ocular health remains unclear, and additional studies are necessary to elucidate its effects.

Furthermore, we were unable to clearly distinguish between microbiota-related effects and direct antimicrobial activity in the observed reduction in ocular surface inflammation; these mechanisms likely contribute differently. Although we made dedicated efforts to separate these factors using an experimental design, the highly integrated nature of the ocular microbiome poses inherent limitations. Consequently, the specific modes and the relative contributions of each mechanism remain unclear.

Given this complexity, we recognize that future research should not only focus on *B. siamensis* but also investigate the broader dynamics of the ocular microbiota as a whole. Consequently, the collective role of these microorganisms in modulating inflammation and immune responses is an important topic for further study. Additionally, exploring the specific mechanisms underlying these effects, such as the identification of antimicrobial substances, is crucial for a better understanding of how the ocular microbiome interacts to influence ocular health.

In conclusion, in this study, we isolated and identified a strain of *B. siamensis* from the mouse ocular surface and systematically evaluated its capacity to inhibit fungal growth and alleviate *Fusarium*-induced keratitis. *B. siamensis* demonstrated multifaceted protective mechanisms. In the in vitro experiments, *B. siamensis* exhibited multifaceted antifungal effects: it directly engulfed *Fusarium* hyphae and secreted antifungal substances. Moreover, it exhibited regulation of the host NF-κB signaling pathway by pre-activating, thereby enhancing ocular surface immunity to a “pre-immune” state that facilitates rapid immune responses. This study experimentally explored, at a preliminary level, the interactions among ocular surface barriers, microbiota, and pathogens.

## Supplementary Material

Supplement 1

Supplement 2

Supplement 3
