# Supplementary material for: Pre-Colonization of Bacillus siamensis on Ocular Surface Mitigates Fusarium keratitis Through Direct Antifungal Activity and Pre-Activation of NF-κB Pathway
Source: Invest Ophthalmol Vis Sci. 2025 Sep 17;66(12):38. doi: 10.1167/iovs.66.12.38 (PMC12449817; doi:10.1167/iovs.66.12.38)
Supplement: Supplement 1 [file iovs-66-12-38_s001.docx]

# Supplement Figure Captions


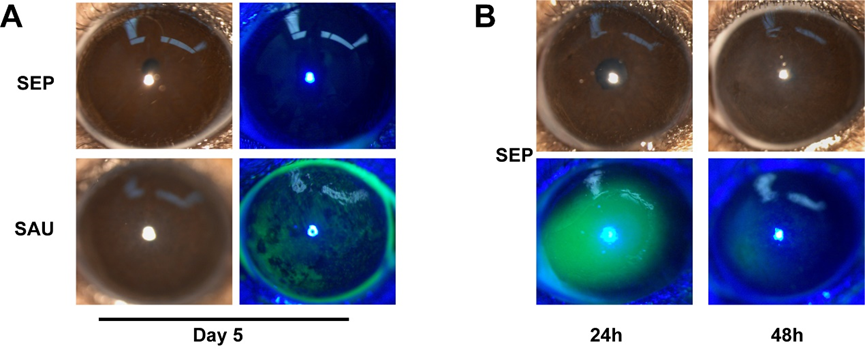


**Figure S1 Safety Assessment of *Staphylococcus aureus* and *Staphylococcus epidermidis* Colonization.** (A) Slit-lamp microscopy and fluorescein sodium staining revealed that *Staphylococcus epidermidis* (SEP) colonization caused no obvious ocular surface damage, whereas *Staphylococcus aureus* (SAU) colonization induced significant inflammatory responses, characterized by corneal opacity and fluorescein retention (n=6). (B) Following corneal epithelial debridement, SEP colonization failed to elicit overt infectious symptoms in the ocular surface (n=6).


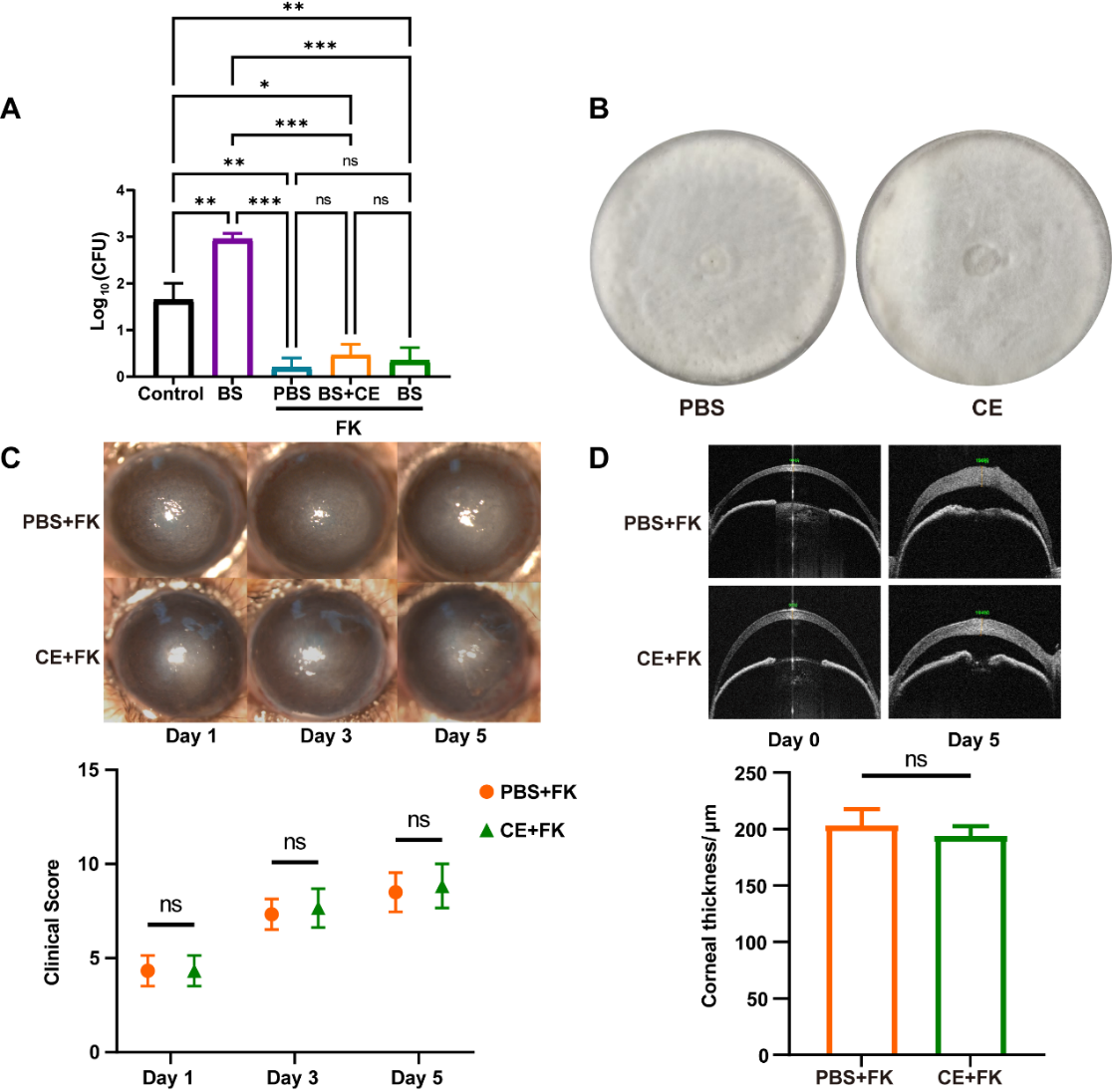


**Figure S2 Pre-colonization with *Bacillus siamensis* Fails to Reduce Its Reduced Abundance during FK.** (A) RT-qPCR analysis of residual *B. siamensis* colonization on the ocular surface after FK (n=3). (B) Agar diffusion assay showed no inhibition zones around agar wells containing crude enzyme solution (CE) (n=3). (C) Slit-lamp microscopy evaluation of the effect of topical CE drops on ocular surface inflammation in FK-infected eyes (CE+FK group vs. PBS+FK group) (n=6). (D) OCT for quantitative analysis of corneal thickness changes.


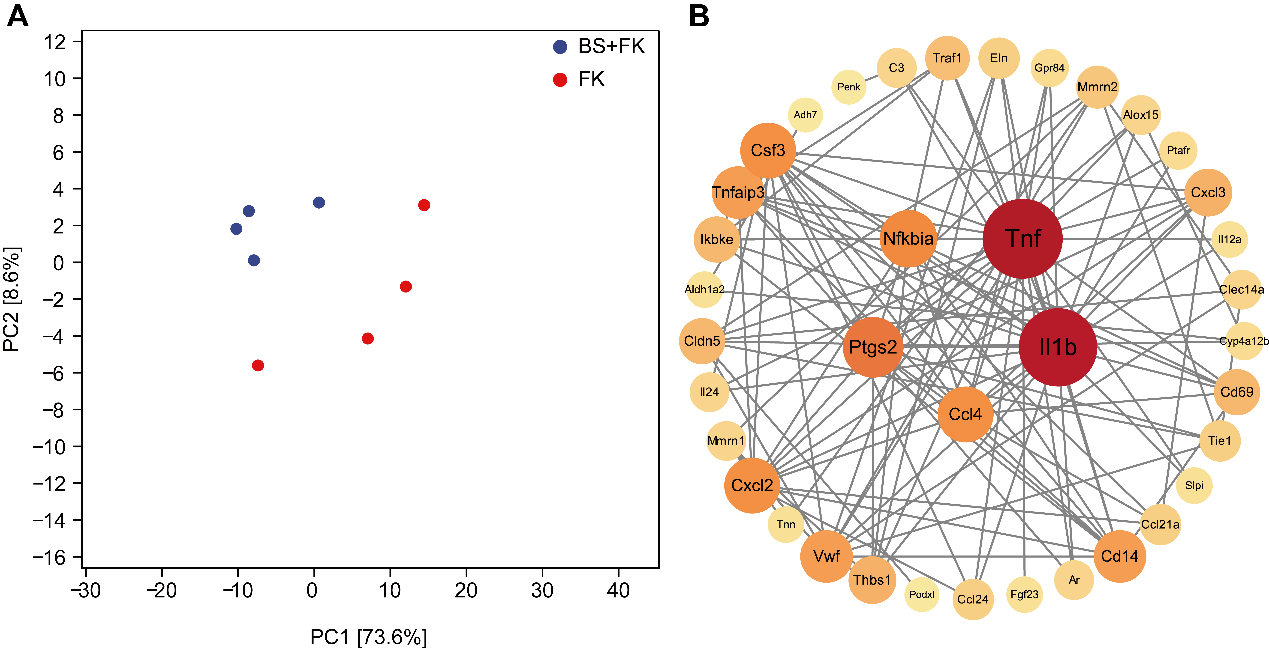


**Figure S3 *B. siamensis* Colonization Alters Gene Expression Profiles in FK.** (A) PCA showing gene expression differences between FK and BS+FK groups. (B) PPI network of differentially expressed genes.


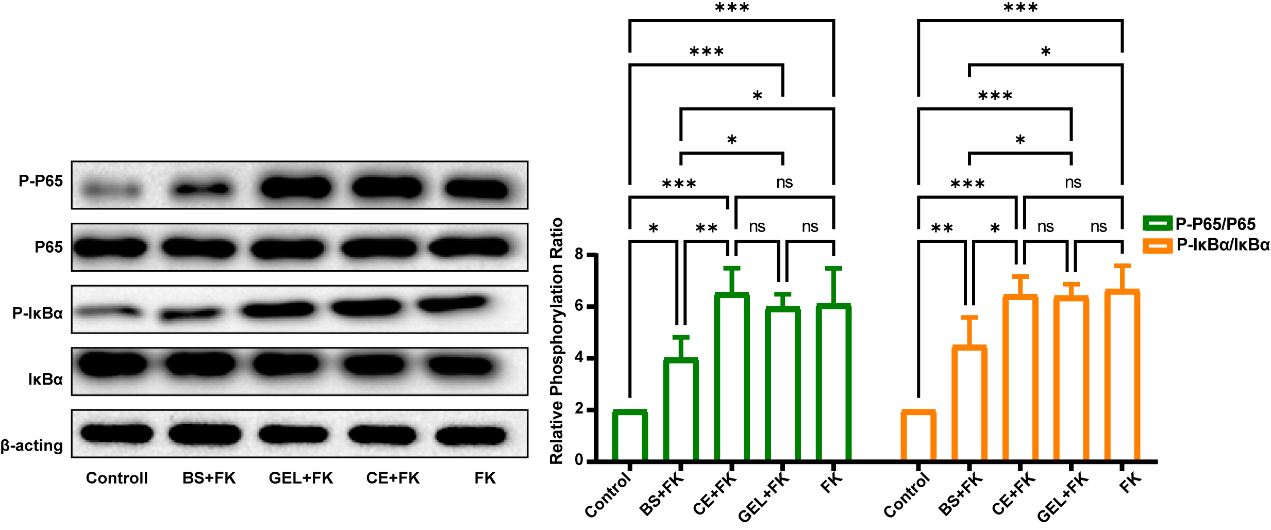


**Figure S4 Colonization Methods does not affect the NF-κB signaling pathway.** The colonization method does not affect the NF-κB signaling pathway. Western blot analysis of protein expression of P-p65, P-IκBα, p65, and IκBα in the GEL+FK (received blank GEL photocrosslinking), CE+FK (treated with crude enzyme solution), BS+FK and control groups, with β-actin serving as a loading control. Densitometric quantification of the relative ratios of p-p65/p65 and p-IκBα/IκBα was performed across all groups (n=3).

**
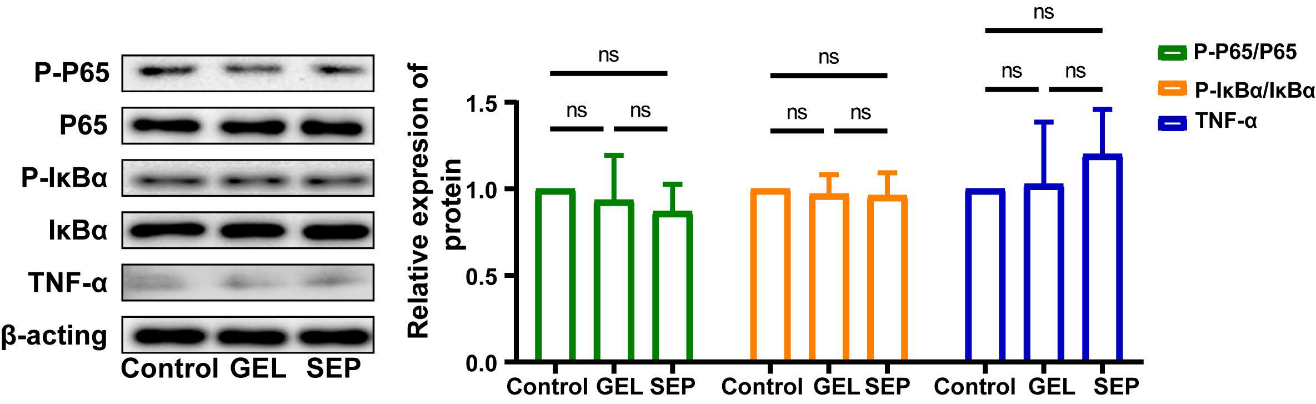
**

**Figure S5 Colonization of *Staphylococcus epidermidis* Exhibits No Significant Activation of NF-κB Pathway.** Western blot was performed to detect the protein expression of p-p65, p-IκBα, p65, and IκBα in the SEP (Colonization of *S. epidermidis*), GEL (received blank GEL photocrosslinking) and control groups (untreated) with β-actin serving as a loading control. Densitometric quantification of the relative ratios of p-p65/p65 and p-IκBα/IκBα was performed across all groups (n=3).


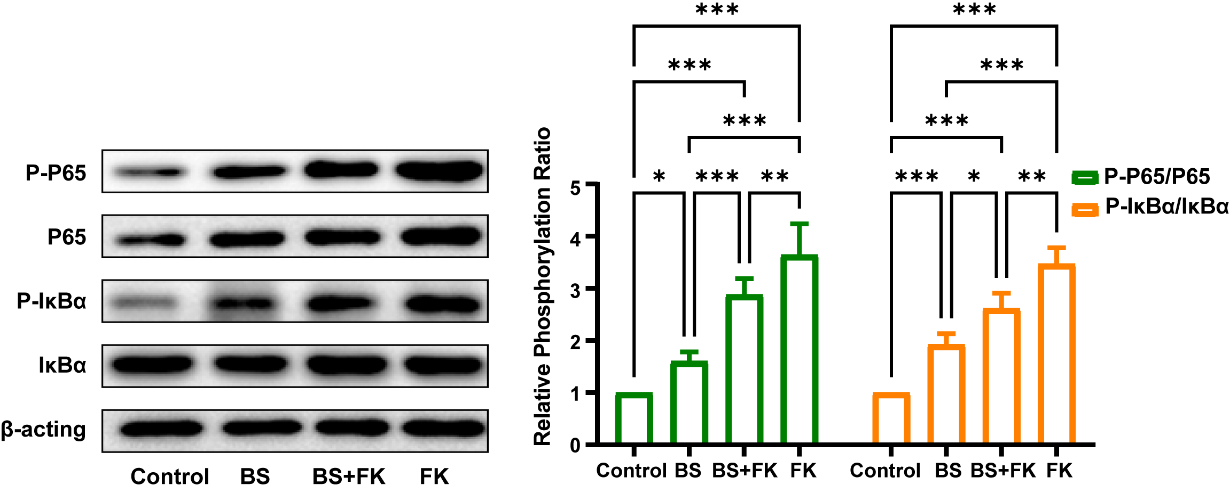


**Figure S6 Overall Evaluation of NF-κB Pathway Activation Across Experimental Groups.** Western blot was performed to detect the protein expression of p-p65, p-IκBα, p65, and IκBα in the Control, FK, BS, and BS+FK groups, with β-actin serving as a loading control. Densitometric quantification of the relative ratios of p-p65/p65 and p-IκBα/IκBα was conducted across all groups (n=4), providing an overall assessment of NF-κB pathway activation status.
