# Supplementary material for: Pre-Colonization of Bacillus siamensis on Ocular Surface Mitigates Fusarium keratitis Through Direct Antifungal Activity and Pre-Activation of NF-κB Pathway
Source: Invest Ophthalmol Vis Sci. 2025 Sep 17;66(12):38. doi: 10.1167/iovs.66.12.38 (PMC12449817; doi:10.1167/iovs.66.12.38)
Supplement: Supplement 2 [file iovs-66-12-38_s002.docx]

**Table S1: Antibodies used in western blot** **and** **Immunofluorescence Staining**

| Target Protein | Antibody Name | Host Species | Dilution Ratio | Catalog Number | Manufacturer |
| --- | --- | --- | --- | --- | --- |
| Phospho-NF-kB p65 | Anti- NF-kB p65 (phospho S536) Antibody | Rabb | 1:1000 | ab76302 | Abcam |
| Phospho-IKB alpha | Anti-IKB alpha (phospho S36) Antibody | Rabbit | 1:1000 | ab133462 | Abcam |
| NF-kB p65 | Anti-NF-kB p65 antibody | Rabbit | 1:1000 | ab32536 | Abcam |
| lkB alpha | Anti-lkB alpha antibody | Rabbit | 1:1000 | ab76429 | Abcam |
| TNF alpha | Anti-TNF alpha antibody Antibody | Rabbit | 1:1000 | ab183218 | Abcam |
| Beta Actin | Anti-beta Actin Antibody | Rabbit | 1:3000 | ab8226 | Abcam |

**Notes:**

- Secondary antibodies (Goat Anti-Rabbit lgG H&L) were used at 1:5000 dilution.
- Membranes were incubated with primary antibodies overnight at 4°C.

**Table S1 Culture Results and 16S Identification**

| **Strain ID** | **Bacterial Strain** | **Frequency** | **Color** | **Source** |
| --- | --- | --- | --- | --- |
| SW1/SW2  /SW3/W4 | Staphylococcus epidermidis | 4 | White/Milky white | Mouse ocular surface |
| Y1/Y2 | Staphylococcus aureus | 2 | Yellow/Golden yellow | Mouse ocular surface |
| G-1 | Bacillus siamensis | 1 | Light yellow or gray | Mouse ocular surface |
| BW-2+BW3 | Bacillus cereus | 2 | White | Mouse ocular surface |
| P1+P2 | Escherichia coli | 2 | Pink | Mouse ocular surface |
| GR | Streptococcus viridans | 1 | greenish | Mouse ocular surface |

**Notes:**

- All screenings were conducted using the modified Gause's No. 1 medium under the conditions of 37°C for 36 hours.

| **GeneID** | **Accession** | **Species** | **Identity** | **Score** | **E_value** | **Annotation** |
| --- | --- | --- | --- | --- | --- | --- |
| GF-TII_TSS20241016-0532-01577-BSND | MN826567.1 | Bacillus siamensis | 1467/1468(99.93) | 2704 | 0 | cqsM9 16S ribosomal RNA gene, partial sequence |
| GF-TII_TSS20241016-0532-01577-BSND | CP053377.1 | Bacillus velezensis | 1467/1468(99.93) | 2704 | 0 | EN01 chromosome, complete genome |
| GF-TII_TSS20241016-0532-01577-BSND | CP053376.1 | Bacillus amyloliquefaciens | 1467/1468(99.93) | 2704 | 0 | WF02 chromosome, complete genome |
| GF-TII_TSS20241016-0532-01577-BSND | CP051463.1 | Bacillus velezensis | 1467/1468(99.93) | 2704 | 0 | UCMB5140 chromosome, complete genome |
| GF-TII_TSS20241016-0532-01577-BSND | CP051011.1 | Bacillus velezensis | 1467/1468(99.93) | 2704 | 0 | BY6 chromosome, complete genome |
| GF-TII_TSS20241016-0532-01577-BSND | LC537275.1 | Bacillus velezensis | 1467/1468(99.93) | 2704 | 0 | B2-9 gene for 16S ribosomal RNA, partial sequence |
| GF-TII_TSS20241016-0532-01577-BSND | MT126339.1 | Bacillus sp. | 1467/1468(99.93) | 2704 | 0 | 84 16S ribosomal RNA gene, partial sequence |
| GF-TII_TSS20241016-0532-01577-BSND | MT114570.1 | Bacillus velezensis | 1467/1468(99.93) | 2704 | 0 | NN04 16S ribosomal RNA gene, partial sequence |
| GF-TII_TSS20241016-0532-01577-BSND | CP031694.1 | Bacillus velezensis | 1467/1468(99.93) | 2704 | 0 | SRCM101368 chromosome, complete genome |
| GF-TII_TSS20241016-0532-01577-BSND | CP028961.1 | Bacillus velezensis | 1467/1468(99.93) | 2704 | 0 | SRCM102752 chromosome, complete genome |
| GF-TII_TSS20241016-0532-01577-BSND | CP028211.1 | Bacillus velezensis | 1467/1468(99.93) | 2704 | 0 | SRCM102747 chromosome, complete genome |
| GF-TII_TSS20241016-0532-01577-BSND | CP028210.1 | Bacillus velezensis | 1467/1468(99.93) | 2704 | 0 | SRCM102746 chromosome, complete genome |
| GF-TII_TSS20241016-0532-01577-BSND | CP028208.1 | Bacillus velezensis | 1467/1468(99.93) | 2704 | 0 | SRCM102744 chromosome, complete genome |
| GF-TII_TSS20241016-0532-01577-BSND | CP028207.1 | Bacillus velezensis | 1467/1468(99.93) | 2704 | 0 | SRCM102743 chromosome, complete genome |
| GF-TII_TSS20241016-0532-01577-BSND | CP028206.1 | Bacillus velezensis | 1467/1468(99.93) | 2704 | 0 | SRCM102742 chromosome, complete genome |
| GF-TII_TSS20241016-0532-01577-BSND | CP028205.1 | Bacillus velezensis | 1467/1468(99.93) | 2704 | 0 | SRCM102741 chromosome, complete genome |
| GF-TII_TSS20241016-0532-01577-BSND | CP047157.1 | Bacillus velezensis | 1467/1468(99.93) | 2704 | 0 | FJAT-45028 chromosome, complete genome |
| GF-TII_TSS20241016-0532-01577-BSND | CP045711.1 | Bacillus velezensis | 1467/1468(99.93) | 2704 | 0 | HN-Q-8 chromosome, complete genome |
| GF-TII_TSS20241016-0532-01577-BSND | CP046386.1 | Bacillus velezensis | 1467/1468(99.93) | 2704 | 0 | GA1 chromosome |
| GF-TII_TSS20241016-0532-01577-BSND | MN417011.1 | Bacillus subtilis | 1467/1468(99.93) | 2704 | 0 | BS31 16S ribosomal RNA gene, partial sequence |

**Table S3: Analysis software version**

| **Software** | **Function** | **Version** |
| --- | --- | --- |
| fastp | Data Filtering | 0.22.0 |
| FastQC | Quality Control | 0.11.9 |
| HISAT2 | Mapping | 2.1.0 |
| HTSeq | Quantitative Analysis of Gene Expression | 0.9.1 |
| DESeq2 | Differential Expression Analysis | 1.38.3 |
| ggplot2 | Volcano / MA | 3.4.4 |
| Mfuzz | Trend | 2.60.0 |
| Circlize | GenomeCircos | 0.4.15 |
| clusterProfiler | Enrichment Analysis | 4.6.0 |
