# Supplementary material for: Pre-Colonization of Bacillus siamensis on Ocular Surface Mitigates Fusarium keratitis Through Direct Antifungal Activity and Pre-Activation of NF-κB Pathway
Source: Invest Ophthalmol Vis Sci. 2025 Sep 17;66(12):38. doi: 10.1167/iovs.66.12.38 (PMC12449817; doi:10.1167/iovs.66.12.38)
Supplement: Supplement 3 [file iovs-66-12-38_s003.docx]

**Eligibility criteria for fungal keratitis patients included in 16S rRNA sequencing**

**Inclusion criteria**

Patients were included if they met any of the following criteria: 1. Fungal hyphae or spores observed by microscopic examination of corneal scraps; 2. Positive fungal culture from corneal scraping specimens; 3. Histopathological confirmation of fungal infection in corneal tissue.

**Exclusion criteria**

Patients were excluded if they met any of the following conditions:1. Use of contact lenses; 2. Bilateral fungal keratitis; 3. Presence of systemic diseases that may interfere with the ocular surface microbiota; 4. administration of eyedrops (antibiotics, corticosteroids, and non-steroidal anti-inflammatory agents) within 6 months.

**High-throughput 16S rRNA sequencing**

**DNA amplification**

After evaluating the quantity and quality of DNA with NanoDrop and agarose gel electrophoresis, samples were loaded onto a 0.6% agarose gel and subjected to 120V constant voltage electrophoresis for 15 minutes. DNA samples with 50 ng of intact or minimally degraded DNA were used for PCR amplification (Cat. No. 580BR10905, Bio-rad, Hercules, CA, USA) after dilution to 1 ng/ml as a template, using primers and Takara Ex Taq (Cat. No. RR001Q, Takara, Shiga, Japan), according to the manufacturer's instructions. The V3-V4 regions of 16S rRNA were amplified using universal primers 343F (5’- TACGGRAGGCAGCAG -3’) and 798R (5’- AGGGTATCTAATCCT-3’).

**Library construction**

Amplicon quality was assessed via gel electrophoresis, then refined using AMPure XP beads (Agencourt), followed by another round of PCR amplification using primers 343F and 798R. After a second purification with AMPure XP beads, the amplicon was quantified using the Qubit dsDNA assay kit (Cat. No. Q32852, Life Technologies, Carlsbad, CA, USA), producing the final amplicon. Equal quantities of refined amplicon were pooled for subsequent high-throughput sequencing (Illumina miseq pe300).

**Bioinformatic analysis**

The raw sequencing data were stored in FASTQ format. Paired-end reads were processed with Trimmomatic software (version 0.53) to remove ambiguous bases (N). Sequences with an average quality score below 20 were trimmed using the sliding window approach in Trimmomatic. After trimming, paired-end reads were merged using FLASH software (version 1.2.11) with parameters set for minimal overlap of 10 bp, a maximum mismatch rate of 20%, and a maximum overlap of 200 bp. Further, sequences were denoised by removing ambiguous, homologous sequences or those shorter than 200 bp. Only sequences with at least 75% of bases scoring above Q20 were retained, and sequences containing chimeras were excluded. These processes were carried out using QIIME software (version 1.8.0). Clean reads underwent primer removal and clustering to define operational taxonomic units (OTUs) with 97% similarity using Vsearch software (version 2.4.2). Representatives from each OTU were selected using the QIIME package. All representative reads were annotated and compared against the Silva database (version 123) and the Greengenes database using the RDP classifier (confidence threshold set to 70%).

A predicted interaction network of the top 30 bacterial genera was constructed based on the Spearman correlation coefficient using the R statistical software (corrplot package). Bacterial genera with |SpearmanCoef| > 0.8 and p < 0.01 were highlighted.

**Parameters of microbial community**

Alpha diversities were used to delineate within-community characteristics. Good’s coverage index reflected the depth of sequencing. The closer the index was to 1, it meant that the depth of sequencing had basically covered all species in the sample. Observed species parametrized the actual number of observed OTUs. Chao1 index estimated how many kinds of OTUs actually presented in the community. Both Shannon Wiener index and Simpson’s index of diversity (1-D) are estimators of species richness and evenness, but the former was more sensitive to species richness, whereas the latter was more sensitive to evenness.^1,2^ The larger the value of phylogenetic diversity index represented the microbial community was constituted by species that had farther relationship of evolution between each other^3^.

**Spore germination inhibition and gram-stained co-culture microscopy**

For spore germination inhibition assays, a Fusarium solani spore suspension (1 × 10⁶ spores/mL) was mixed with the *Bacillus siamensis* enzyme solution at a 1:1 volume ratio. The mixture was incubated at 36°C for 24 h, and spore germination was assessed microscopically.

For the co-culture interaction assay, a groove was made in PDA medium and covered with a sterile coverslip. F. solani and B. siamensis were inoculated on opposite sides of the groove and incubated at 27°C for 48 h. The coverslips were transferred to glass slides, fixed, and subjected to Gram staining using a commercial kit (Solarbio, Beijing, China). Microscopic imaging was performed using an Eclipse E800 microscope (Nikon, Tokyo, Japan).

**Preparation protocol for the ocular surface transient crude enzyme solution**

To prepare a crude enzyme solution for restoring the BS-colonized ocular environment following fungal keratitis modeling, conjunctival swabs were collected from BS-colonized mice on Day 1 after colonization. Swabs were vortexed in 0.5 mL PBS to release microorganisms. The suspension was mixed with 4.5 mL of LB broth and pre-cultured at 37°C for 6 hours to amplify the ocular surface microbiota.

Subsequently, 0.5 mL of Fusarium solani spore suspension in PBS (1 × 10⁶ spores/mL) was added to the culture and co-incubated at 37°C for an additional 12 hours. After incubation, the supernatant was collected by centrifugation and filtered through a 0.22 μm sterile membrane to obtain the crude enzyme solution.

Based on the results of the CCK-8 assay, the crude enzyme solution was diluted with PBS to prepare a 5% eye drop formulation, which was used to compensate for microbiota disruption caused by FK modeling.

**Cell culture**

Human SV40-immortalized corneal epithelial cells (CRL-11135, HCE-2, ATCC, USA) were cultured in DMEM/F12 medium (Gibco, USA) supplemented with 10% fetal bovine serum (FBS; Gibco, USA) and maintained in a humidified incubator at 37°C with 5% CO₂. The culture medium was replaced every 2–3 days.

**Bacterial Colonization via GelMA Carrier**

The GelMA hydrogel matrix was prepared by dissolving 60% GelMA (Engineering for Life, Jiangsu, China) in sterile PBS containing 0.25% lithium phenyl-2,4,6-trimethylbenzoylphosphinate (LAP) as a photoinitiator. The solution was sterilized by filtration through a 0.22 μm membrane filter and stored at 37°C to prevent premature gelation.

Separately, a bacterial suspension of *B. siamensis* was prepared by harvesting cells at mid-log phase and adjusting the concentration to 1×10⁸ CFU/mL in sterile PBS. The bacterial suspension was then mixed with the pre-warmed, sterilized GelMA-LAP solution to form the bacterial-GelMA composite hydrogel, which was maintained at 37°C until use.

A 5-μL droplet of the bacterial-GelMA suspension was applied to the ocular surface and photo-crosslinked using a 405 nm light source at 5 mW in a point irradiation mode. The light source was positioned horizontally to the corneal surface to minimize exposure to surrounding tissues and ensure targeted irradiation of the gel for 5–20 seconds.

**Isolation of *Bacillus siamensis***

This modified Gause's No. 1 medium was originally designed to selectively isolate Actinomycetes, which were identified through sequencing in our previous studies.^4,5^ Actinomycetes, known for their probiotic properties, can secrete antimicrobial substances and are among the primary sources of antibiotics.^6,7^ These bacteria play a significant role in the natural microbial balance and offer protection against pathogenic organisms. However, through this screening method, we unexpectedly isolated *B.siamensis* instead. The formulation of the culture medium used in this experiment is as follows.

**Translation of the Medium Composition (g/L):**

- Potassium Nitrate (KNO_3_) - **1.0**
- Dipotassium Hydrogen Phosphate (K_2_HPO_4_) - **0.5**
- Magnesium Sulfate (MgSO_4_) - **0.5**
- Ferrous Sulfate (FeSO_4_) - **0.01**
- Sodium Chloride (NaCl) - **0.5**
- Soluble Starch - **20.0**
- Casein - **15.0**
- **pH 7.2 – 7.4**

**RNA Sequencing and Analysis**

RNA sequencing and subsequent bioinformatics analysis were performed by Personal Biotechnology (Shanghai, China). Briefly, total RNA was isolated using Trizol Reagent, and its concentration, quality, and integrity were assessed using a NanoDrop spectrophotometer (Thermo Scientific). Sequencing libraries were prepared from 3 µg of total RNA, with mRNA purification using poly-T oligo-attached magnetic beads, fragmentation, and cDNA synthesis. Libraries were sequenced on the NovaSeq 6000 platform (Illumina, San Diego, CA, USA) to generate paired-end reads. Raw data were filtered to obtain high-quality clean reads, which were then aligned to the reference genome using HISAT2. Gene expression levels were quantified using HTSeq and normalized to FPKM/TPM.

Differential gene expression analysis was performed using DESeq2 with thresholds of |log2FoldChange| > 1 and adjusted p-value < 0.05. Functional enrichment analysis, including Gene Ontology (GO) and Kyoto Encyclopedia of Genes and Genomes (KEGG) pathways, was conducted using ClusterProfiler. For software versions, refer to **Supplement Table 3**.

**Supplement References**

1. Hill TC, Walsh KA, Harris JA, Moffett BF. Using ecological diversity measures with bacterial communities. FEMS Microbiol Ecol. Feb 1 2003;43(1):1-11. doi:10.1111/j.1574-6941.2003.tb01040.x

2. Kim BR, Shin J, Guevarra R, et al. Deciphering Diversity Indices for a Better Understanding of Microbial Communities. J Microbiol Biotechnol. Dec 28 2017;27(12):2089-2093. doi:10.4014/jmb.1709.09027

3. Faith DP, Baker AM. Phylogenetic diversity (PD) and biodiversity conservation: some bioinformatics challenges. Evol Bioinform Online. Feb 17 2007;2:121-128.

4. Ren Z, Liu Q, Li W, Wu X, Dong Y, Huang Y. Profiling of Diagnostic Information of and Latent Susceptibility to Bacterial Keratitis From the Perspective of Ocular Bacterial Microbiota. Front Cell Infect Microbiol. 2021;11:645907. doi:10.3389/fcimb.2021.645907

5. Ren Z, Li W, Liu Q, Dong Y, Huang Y. Profiling of the Conjunctival Bacterial Microbiota Reveals the Feasibility of Utilizing a Microbiome-Based Machine Learning Model to Differentially Diagnose Microbial Keratitis and the Core Components of the Conjunctival Bacterial Interaction Network. Front Cell Infect Microbiol. 2022;12:860370. doi:10.3389/fcimb.2022.860370

6. Yi R, Shi Y, Cao X, Pan C. Actinomycetes: Treasure trove for discovering novel antibiotic candidates. Eur J Med Chem. Mar 15 2025;286:117317. doi:10.1016/j.ejmech.2025.117317

7. Schniete JK, Fernandez-Martinez LT. Natural product discovery in soil actinomycetes: unlocking their potential within an ecological context. Curr Opin Microbiol. Jun 2024;79:102487. doi:10.1016/j.mib.2024.102487
